# Seasonal calving in European Prehistoric cattle and its impacts on milk availability and cheese-making

**DOI:** 10.1038/s41598-021-87674-1

**Published:** 2021-04-14

**Authors:** Marie Balasse, Rosalind Gillis, Ivana Živaljević, Rémi Berthon, Lenka Kovačiková, Denis Fiorillo, Rose-Marie Arbogast, Adrian Bălăşescu, Stéphanie Bréhard, Éva Á. Nyerges, Vesna Dimitrijević, Eszter Bánffy, László Domboróczki, Arkadiusz Marciniak, Krisztián Oross, Ivana Vostrovská, Mélanie Roffet-Salque, Sofija Stefanović, Maria Ivanova

**Affiliations:** 1 UMR 7209 AASPE « Archéozoologie, archéobotanique: sociétés, pratiques, environnements » CNRS, MNHN, 75005 Paris, France; 2 BioSense Institute, University of Novi Sad, 21000 Novi Sad, Serbia; 3 Laboratory of Archaeobotany and Palaeoecology, Faculty of Science, University of South Bohemia, 37005 České Budějovice, Czech Republic; 4 UMR 7044 “Archimède”, MISHA, CNRS, University of Strasbourg, 67000 Strasbourg, France; 5 Department of Bioarchaeology, “Vasile Pârvan” Institute of Archaeology, Romanian Academy, 010667 Bucharest, Romania; 6 Department of Archaeology, Savaria Museum, Szombathely, 9700 Hungary; 7 Laboratory for Bioarchaeology, Department of Archaeology, Faculty of Philosophy, University of Belgrade, 11000 Belgrade, Serbia; 8 Römisch-Germanische Kommission des Deutschen Archäologischen Instituts, 60325 Frankfurt/Main, Germany; 9 Dobó István Castle Museum, Vár út 1, 3300 Eger, Hungary; 10 Faculty of Archaeology, Adam Mickiewicz University, 61-614 Poznań, Poland; 11 Institute of Archaeology, Research Centre for the Humanities, Eötvös Loránd Research Network, Centre of Excellence of the Hungarian Academy of Sciences, 1097 Budapest, Hungary; 12 Institute of Archaeology and Museology, Masaryk University, 60200 Brno, Czech Republic; 13 Department of History, Palacký University, 77900 Olomouc, Czech Republic; 14 Organic Geochemistry Unit, School of Chemistry, University of Bristol, Bristol, BS8 1TS UK; 15 Vienna Institute for Archaeological Science (VIAS), University of Vienna, 1190 Vienna, Austria; 16 Institut Für Ur- und Frühgeschichte und Vorderasiatische Archäologie, Universität Heidelberg, 69117 Heidelberg, Germany; 17 Present Address: Interdisciplinary Center for Archaeology and Evolution of Human Behaviour (ICArEHB), Faculdade de Ciências Humanas e Sociais, Universidade do Algarve, 8005-139 Faro, Portugal

**Keywords:** Biogeochemistry, Environmental social sciences

## Abstract

Present-day domestic cattle are reproductively active throughout the year, which is a major asset for dairy production. Large wild ungulates, in contrast, are seasonal breeders, as were the last historic representatives of the aurochs, the wild ancestors of cattle. Aseasonal reproduction in cattle is a consequence of domestication and herding, but exactly when this capacity developed in domestic cattle is still unknown and the extent to which early farming communities controlled the seasonality of reproduction is debated. Seasonal or aseasonal calving would have shaped the socio-economic practices of ancient farming societies differently, structuring the agropastoral calendar and determining milk availability where dairying is attested. In this study, we reconstruct the calving pattern through the analysis of stable oxygen isotope ratios of cattle tooth enamel from 18 sites across Europe, dating from the 6th mill. cal BC (Early Neolithic) in the Balkans to the 4th mill. cal BC (Middle Neolithic) in Western Europe. Seasonal calving prevailed in Europe between the 6th and 4th millennia cal BC. These results suggest that cattle agropastoral systems in Neolithic Europe were strongly constrained by environmental factors, in particular forage resources. The ensuing fluctuations in milk availability would account for cheese-making, transforming a seasonal milk supply into a storable product.

## Introduction

Control over reproduction has become one of the most important factors for enhancing production in present-day industrial husbandry. Although selection is a long-standing practice in the history of domestication^1^, total control over animal reproduction only developed recently. Artificial insemination combined with gene selection is the latest extension of the marked intensification of the artificial selection of animals, initiated in the eighteenth century. Another dimension of the control exerted on domestic animal reproduction is the regulation of fertility cycles. The extent to which this type of control may have been applied in early farming societies in pre- and protohistoric times has been debated, in particular in relation to reproduction seasonality in domestic stock and its consequences on the subsistence economy and related social organization^2–6^). Farm mammals have inherited remnants of a seasonal reproduction strategy from their wild ancestors^7^. By providing protection against environmental constraints, domestication led to the attenuation of some of the physiological expressions of seasonality, but to varying extents among species. Modern domestic pigs generally breed throughout the year, but many of them manifest seasonal fertility impairment^8^. Domestic sheep and goats in temperate latitudes still express genuine periodic anoestrus, with strong consequences on the seasonal availability of animal products^9^. In contrast, modern domestic cattle are aseasonal breeders: cows are reproductively active throughout the year enabling year-round or seasonal breeding through livestock management, depending on local conditions and the orientation of production. Today, aseasonal breeding in cattle is a major asset for dairy production, as the milk supply is regulated by the calving season.

Aseasonal reproduction in cattle is a direct consequence of domestication. Taurine cattle (*Bos taurus*) descend from the aurochs (*Bos primigenius*), domesticated in the Middle Euphrates Valley in Southwest Asia in the ninth millennium BC^10^. Although the reproductive pattern of the prehistoric aurochs is not directly documented, seasonal breeding behaviour is very likely in this large ungulate. Observations by sixteenth-century naturalists on the last remnant aurochs population in Poland suggest that reproduction was restricted to limited periods of the year, with a short regular mating season in late summer and calving in late spring^11,12^. Cows and immature bulls lived separately from older bulls, and only joined them briefly during the mating season, which is characteristic of species with pronounced sexual dimorphism^12^.

Exactly when the capacity for aseasonal breeding evolved in domestic cattle remains unknown. It has been suggested that the aurochs could have had the latent capability to breed throughout the year^13^. In domestic cattle, the reduction of sexual dimorphism and herding males and females together could have favoured the onset of aseasonal breeding^13^. Conditions could therefore have been propitious to aseasonal breeding since prehistoric times. However, year-round breeding would also require overcoming environmental constraints, especially fluctuations in food availability. Nutrition is an important factor in cattle reproductive performances. Unmanaged herds of primitive breeds kept in nature reserves in Europe exhibit seasonal reproduction in synchrony with vegetation dynamics (Supplementary Table S1). The white cattle of Chillingham Park in Northern England, which breed year-round despite strong seasonality in climate and vegetation dynamics^14^, are an exception. Consequently, it appears that even in present-day unimproved cattle breeds, the physiological capacity for year-round breeding is rarely expressed when environmental conditions are not conducive to the supply of an adequate quality and quantity of food throughout the year.

The breeding pattern in domestic cattle therefore depends on the combination of three interrelated factors—physiological capacity for aseasonal breeding, management strategy for males and females, and the availability of food resources. When the precondition of physiological capacity for aseasonal breeding is fulfilled, the herder can choose between the practices of year-round or seasonal breeding. In traditional herding systems, keeping males and females together throughout the year can result in year-round breeding. The main advantage of this practice is continuous milk supply for the household, but it presupposes good nutritional level throughout the seasons to ensure regular fertility. Conversely, keeping males separately for part of the year will result in seasonal breeding. This second option allows the herder to schedule the calving season to the period when food resources are most reliable and to limit the duration of tasks devoted to cow and calf care. However, it requires careful management of the interactions between males and females to enable successful mating. In both cases, the availability of food resources throughout the year greatly influences management decisions.

In view of the above, it could be expected that in the past, cattle raised outdoors with limited complementary feed would exhibit seasonal reproductive behaviours^2^. In this regard, investigations into cattle birth seasonality in archaeological sites from diverse chronocultural complexes have led to different conclusions. Studies involving stable isotope analysis in tooth enamel, conducted on sites dated to the early sixth to fourth millennia cal BC across Europe, have revealed either a restricted birth period^2,15–21^, or multiple birth seasons^5^. Multiple-season calving, perhaps even year-round calving, was also observed at more recent sites dated to the Early Bronze Age and Iron Age in the British Isles^3,4^. Seasonal or aseasonal calving would have affected the economic and social components of ancient farming societies differently, determining the availability of cattle milk throughout the year, structuring the agropastoral calendar, scheduling herd mobility in mountainous areas^22,23^, and generally shaping farmers’ lives. In this study, we present a new European-scale dataset of stable oxygen isotope measurements in tooth enamel, enabling the reconstruction of the calving pattern. Considering that milk was exploited by prehistoric farming societies at that time and earlier^24–31^, how the calving pattern impacted the seasonal availability of milk and ultimately cheese making is also discussed.

## Sites, cultural complexes and climatic framework

**Figure 1:**
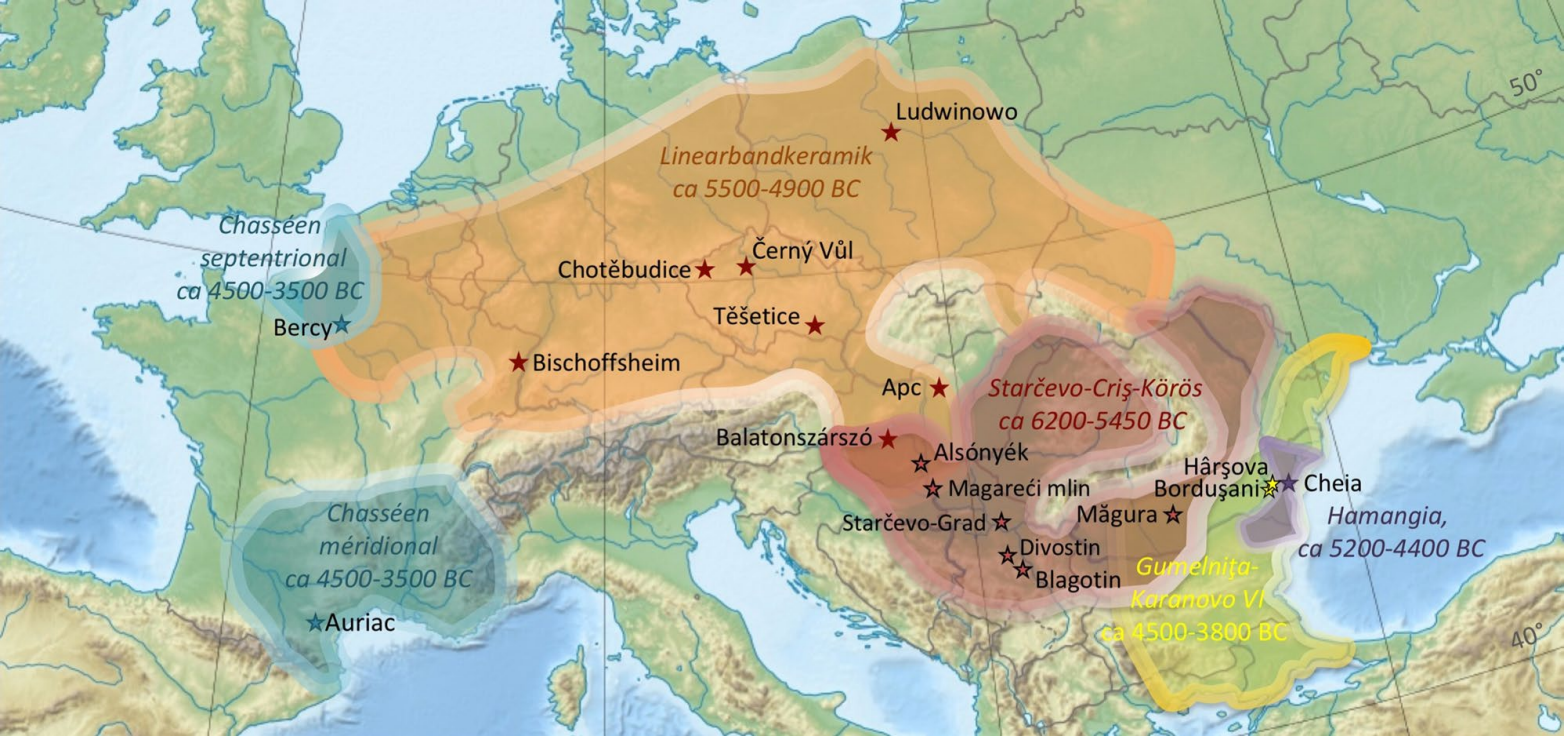
Location of the sites included in the study and their cultural affiliation (Europe relief map by Alexrk2, CC BY-SA 3.0 < https://creativecommons.org/licenses/by-sa/3.0 > , via Wikimedia Commons; https://upload.wikimedia.org/wikipedia/commons/7/79/Europe_relief_laea_location_map.jpg; distribution of regional cultures after^32,80–82^; the LBK complex here includes the Alföld LBK).

The sample includes 119 animals from 18 sites across the European continent and encompasses a time scale from the Balkans Early Neolithic to the Middle Neolithic in Western Europe. The sites (Fig. 1; see site description in Supplementary Note S1 and dates in Supplementary Table S2) belong to the Starčevo-Çris-Körös complex, pioneer farmers who settled in the interior of the Balkans and on the southern fringes of the Great Pannonian Plain by the turn of the sixth millennium cal BC; the Linearbandkeramik complex (LBK, ca 5500–4900 cal BC), or the earliest Neolithic in Central Europe; the Hamangia culture or early Chalcolithic communities settled in the Central Dobruja plateau, west of the Black Sea and east of the Danube, dated to the fifth millennium cal BC and contemporaneous with the latest stages of the LBK; the Gumelniţa culture, or later developments of the Chalcolithic in Dobruja, characterized by permanent settlement which probably marked a rupture in social and economic systems^32,33^; and the Chasséen cultural complex in Western Europe, dated to the beginning of the fourth millennium cal BC. Cattle played important economic and social roles in all these cultural complexes. Compared to the earliest Neolithic societies in the Aegean and in the southern Balkans, where ovicaprids (sheep/goats) were massively predominant, cattle played an increased economic role in the Starčevo-Çris-Körös communities of the northern Balkans and Carpathian Basin^28,34^. They were also part of ritual activities, as shown by a newly identified large-horned clay cattle figurine, which sheds light on the role of cattle in domestic rituals^35^. Later on, in the LBK sites of Central Europe, cattle are usually abundantly represented and the analysis of mortality profiles suggests that milk exploitation was a widespread practice^30^. In the Romanian Chalcolithic, cattle clearly predominated the economy in Hamangia societies^36^, while their representation diminished in the faunal assemblages of the Gumelniţa culture, sometimes outnumbered by ovicaprids or pig^33^. Cattle also played a predominant role in the economies of the southern/northern Chasséen communities in France, where they were exploited for milk and meat^37^.

Eastern and Central Europe (including eastern Germany) are predominantly under a humid continental climate with warm to hot summers, cold winters (freezing average temperature in the coldest month and at least four months below 10 °C) and no significant difference in precipitation average between seasons and snowfall in winter (Dfb type climate, according to the Köppen-Geiger climate classification;^38^). The Black Sea border in southeastern Romania is influenced by a steppic climate (or cold semi-arid, Bsk type) with cold snowy winters and hot dry summers. By comparison, Western Europe has a temperate oceanic climate (Cfb type) with at least one snowfall in winter in spite of milder temperature (average temperature in the coldest month above 0 °C), cool summers, constant and reliable precipitation throughout the year. The Mediterranean margin in southern France has a warm summer Mediterranean climate (Cs type) with mild winters and warm dry summers; precipitations are unevenly distributed and occur mainly in winter with snowfall happening in chilly winters. Consequently, all areas are characterized by distinct seasons with large seasonal temperature differences, chilly to cold winters with occasional to regular snowfall. Cold winter temperatures and snow cover would probably most strongly impact the seasonal availability of resources. In some areas, including eastern Romania and the southernmost part of France, the scarcity of rain in summer would also impact forage quality and quantity.

## Principles

Past cattle birth seasonality is investigated through the reconstruction of the seasonal cycle record in molars, using the sequential analysis of the stable oxygen isotope composition (δ^18^O) of enamel mineral fraction (bioapatite). Bioapatite precipitates in oxygen isotopic equilibrium with body water, offset by temperature-dependent fractionation held constant in homeothermic mammals^39^. The δ^18^O value of body water is fundamentally determined by oxygen input and output fluxes. In cattle under grazing conditions, drinking water and feed moisture largely predominate the oxygen input flux^40^. In outdoor conditions, both drinking water and plant water are related to precipitation, explaining why δ^18^O values in animal skeletons linearly correlate to the δ^18^O value in local annual precipitation^41,42^. In continental Europe, the δ^18^O values of precipitation are seasonally affected by variations in air temperature: the highest δ^18^O values are recorded during the warmest months and the lowest during the coldest months^43^, resulting in cyclical variations on an annual scale. Moreover, animal behaviour and physiology in response to changes in temperature and air humidity have also been shown to explain a significant part of seasonal variation in body water δ^18^O value. These external and internal factors interact in a complex manner, resulting in clear temporal annual variations^40^. Seasonal variations in body water δ^18^O values are recorded in tooth δ^18^O values during mineralization and can be reconstructed using a sequential sampling procedure on enamel. As the tooth growth timing is fixed within species, the season of birth determines the sequence of the annual cycle recorded in a given tooth^44–46^. Variability in the birth season is described through the comparison of the position of the maximum value of the δ^18^O cycle (δ^18^O_max_) in the tooth crown. A quantitative estimation of inter-individual variability in the birth season involves modelling the cyclical changes in δ^18^O sequences and normalizing the distances (^47^; see “Material and methods” section).

The third molar (M3) was selected for sampling. As crown development is not completed until the age of two years old for this tooth^48^, a consequence of this sampling strategy is that birth seasonality is inferred from a subsample of the population composed of individuals that survived at least until the age of two. Yet, calf mortality within the first year of life can be high. In the Chillingham cattle breeding throughout the year, Hall & Hall^14^ reported only 50% of calves reaching maturity, with most of this mortality occurring within the first year. Calf mortality was largely attributable to bad weather. If mortality were higher in calves born out of season, as a result of bad weather or scarcity of feeding resources, relying on results obtained from the M3 alone could cause out-of-season births to be overlooked. To check for a bias in the estimation of the distribution of births due to the absence of calves under two years old in the M3 samples, birth seasonality was also investigated in the fourth deciduous premolar (dP4) and the first molar (M1) at Cheia. In both teeth, the crown is partly mineralized in utero, partly within the first months of life^48,49^. We hypothesise that if the results obtained from the M3 are biased due to higher mortality in calves born out of season, then the record in the dP4 and M1 would show greater variability in the season of birth than observed in the M3.

## Results

**Figure 2:**
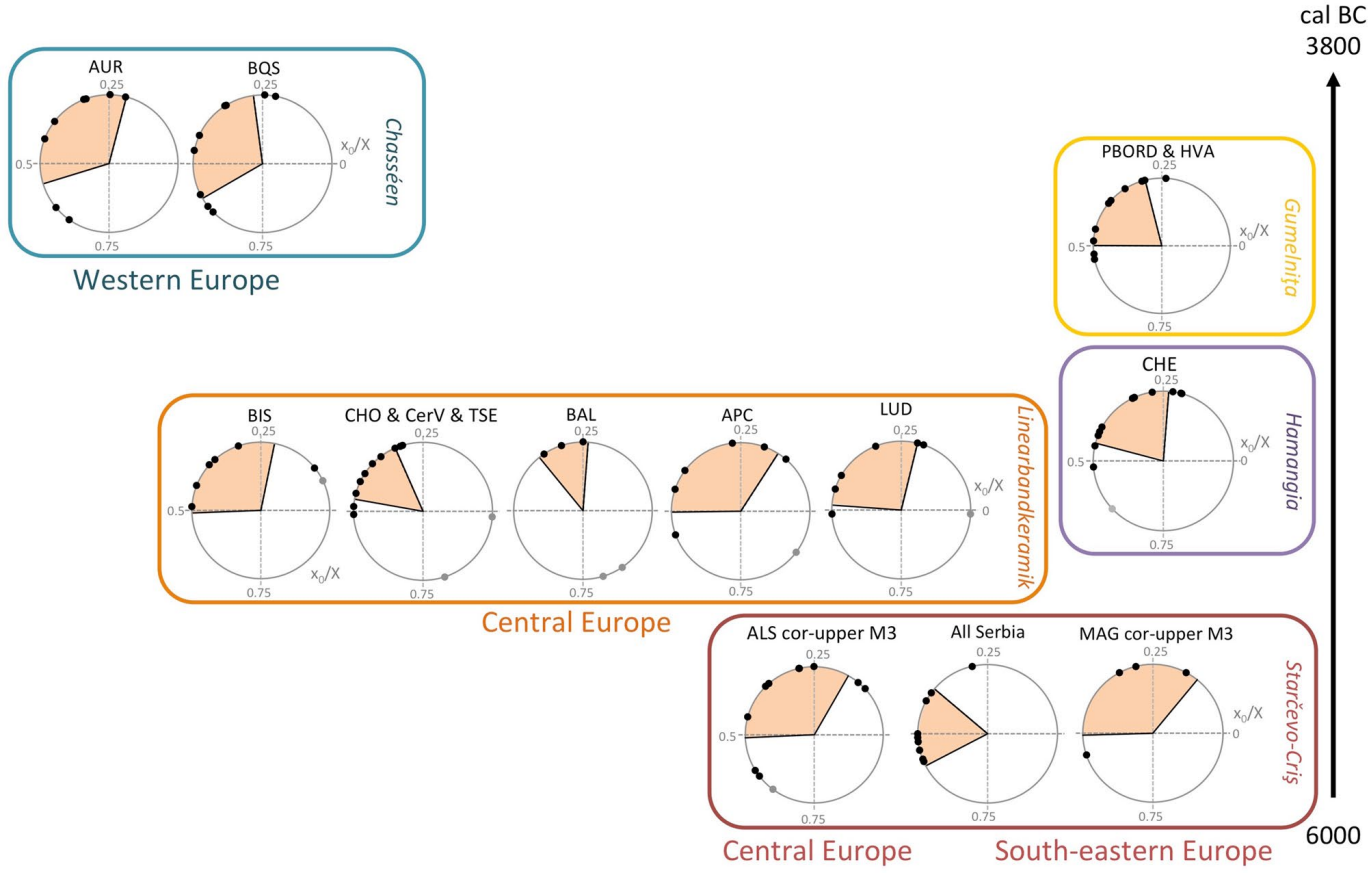
Distribution of cattle births throughout the annual cycle. The x_0_/X ratio refers to the normalized location of δ^18^O_max_ in the tooth crown (see Methods). All data from lower M3, unless specified. Results from upper M3 are corrected for the purposes of comparison with the lower M3s (“cor-upper M3” see Methods). Confidence interval (shaded 68%) for the period of births; outliers (outside 80%; •) were not considered in these statistics. All Serbia = Starčevo-Grad (data : this study), Magareći mlin (this study), Divostin (this study), Blagotin (this study); CHO: Chotěbudice^18^; CerV: Černý Vůl^18^; TSE: Těšetice-Kyjovice (this study); LUD: Ludwinowo^19^; BIS: Bischoffsheim (this study and^21^); APC: Apc (this study); BAL: Balatonszárszó-Kis-erdei-dűlő (this study); CHE: Cheia (^17^ and this study); PBORD: Borduşani (^83,84^ and this study); HVA: Hârşova (^84^ and this study); BQS: Bercy (^15^ and this study); AUR: Auriac (this study); MAG: Măgura^16^; ALS: Alsónyék (this study). On the graph presenting birth distributions at (CHO & CerV & TSE) all out-of-season births were found at CHO.

**Figure 3:**
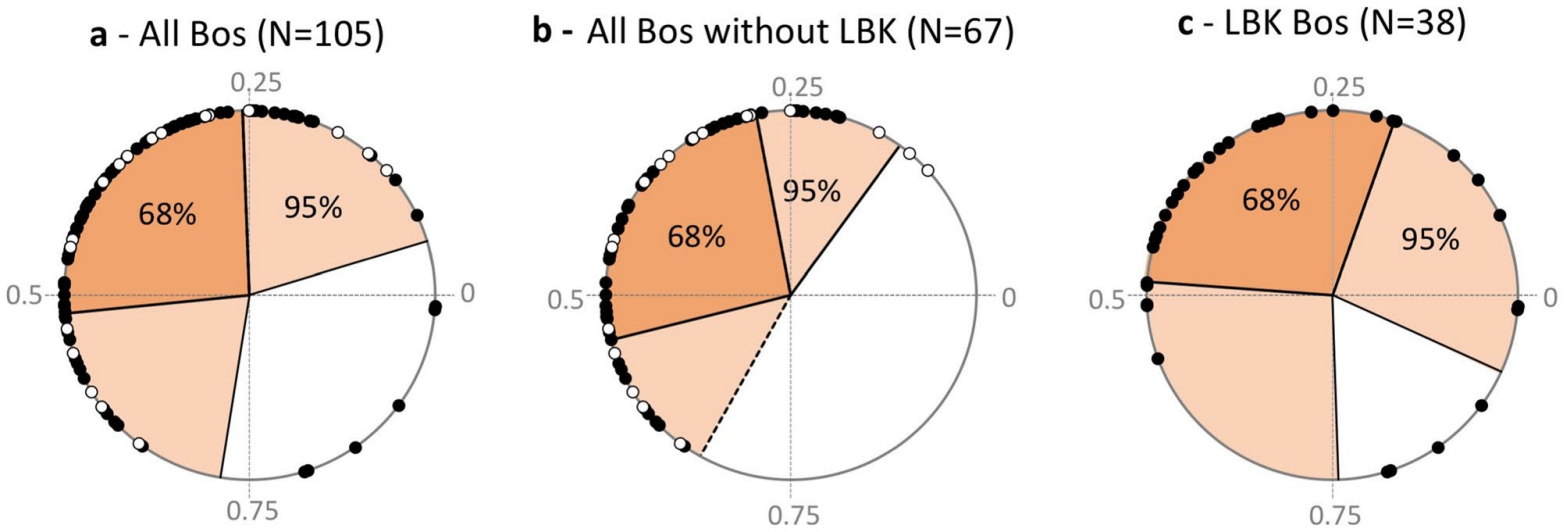
Distribution of cattle births throughout the annual cycle, from the sequence of δ^18^O values measured in the third molar (M3). (**a**) All European sites; (**b**) European sites without Linearbandkeramik sites; (**c**) Linearbandkeramik sites only. The a- and b- datasets include lower (•) and upper third molars (o). Data from the upper M3 were corrected for the purposes of comparison with the lower M3. Only lower molars are included in the statistics. The dark shaded area represents the main birth period (when 68% of births occur); births outside the 95% interval are out-of-season births.

**Figure 4:**
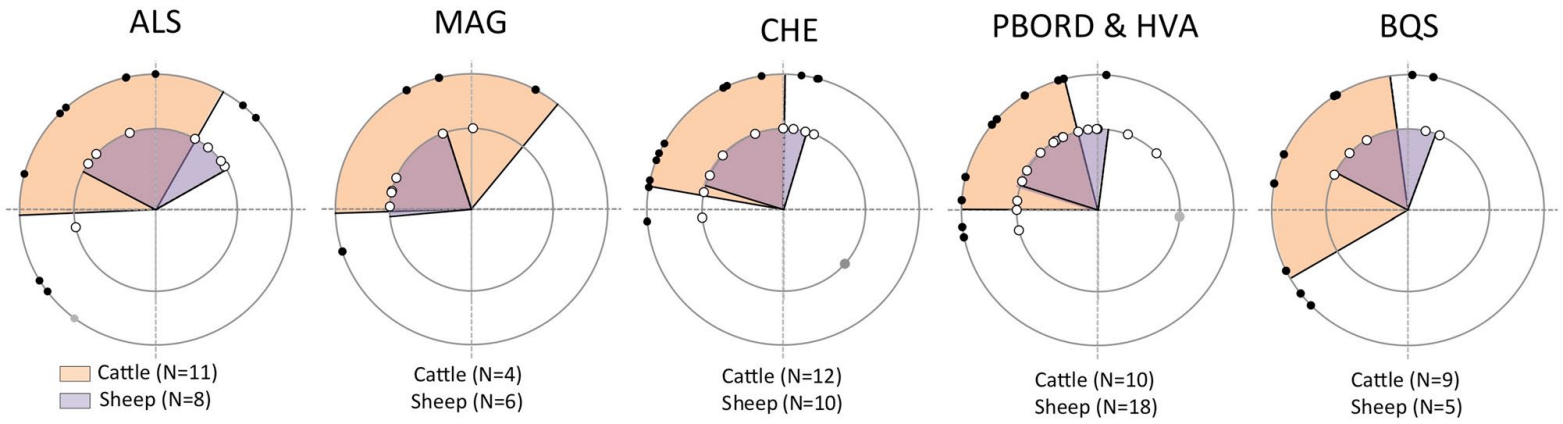
Distribution of cattle and sheep births throughout the annual cycle. CHE: Cheia (sheep data in^85^); PBORD & HVA: Borduşani and Hârşova (sheep data in^84^); BQS: Bercy (sheep data in^15^); MAG: Măgura (cattle: corrected data from upper molars; sheep data in^16^), ALS: Alsónyék (cattle: corrected data from upper molars; sheep data in^84^). Note that the time of the year when calving and lambing occurred may not be directly compared on these graphs. BQS sheep data on M2; all others: data on M3.

Results from the sequential analysis of enamel δ^18^O values from dp4 and M1 at Cheia are shown in supplementary Data S1, Fig. S8 and Table S11. Results from the sequential analysis of enamel δ^18^O values from M3 are shown in Supplementary Tables S3–S9 and Supplementary Figs. S1–S7. Results from the δ^18^O sequences modelling are shown in Supplementary Table S10. Figures 2, 3 and 4 show the distribution of births throughout the annual cycle, as reflected by the position of the δ^18^O sequence optimum in the tooth crown, normalized to the period of the annual cycle in each tooth (x_0_/X) (see “Material and methods” section).

On a site scale, the main calving period is limited to a restricted length of time (from 2.5 months at Borduşani to 4.1 months at Alsónyék and 4.4 months at Măgura, albeit based on a small sample). This is also the case on the regional scale (1.9 months in Czech Republic sites—including Chotěbudice, Černý Vůl and Těšetice-Kyjovice; 2.3 months in Serbian sites including Starčevo-Grad, Magareći mlin, Divostin and Blagotin) (Fig. 2). Cattle births on a geographical and chronological scale fall within the same segment of the year (within the quarter defined by x_0_/X = 0.25 to 0.50, or slightly earlier or later; Fig. 2). On a European scale, most cattle births (68%) occurred within 3.1 months and almost all (95%) over 8.1 months (Fig. 3a). A minimal number of births occurred over the remaining period of 4 months. Within this period of minimal calving, all recorded births belong to LBK sites (Fig. 3). Excluding LBK sites, the duration of the main birth period remains 3.1 months (68% of births), but almost all births occur (95%) occur over only 6.2 months (Fig. 3b). Considering LBK sites only, the main period of births (68%) extends over 3.5 months with 95% of births occurring over 9.9 months (Fig. 3c). It must be noted that all figures given for the length of the birth period are indicative as inter-individual variability in the third molar growth timing could impact the time resolution (see “Material and methods” section).

At Cheia, the dP4 and M1 of calves that died within their first year show very similar patterns of variations in δ^18^O values, suggesting that they were born within the same period of the seasonal cycle (Supplementary Data S1). At this site, this result refutes the hypothesis that the seasonal calving inferred from the third molar dataset is artificially created by a higher mortality rate in calves born out of season.

## Discussion

### Markedly seasonal calving

The identification of a birth season of three months (68% of births) at most of the sites and the overall similarity in the calving period in 18 sites across time and space throughout Europe suggest seasonal calving. The consistency of these results contrasts with multiple season calving reported at the Funnel Beaker (3950–3500 cal BC) site of Almhov in Sweden^5^. At that site, although three dP4 provided δ^18^O sequences showing very similar trends, the authors concluded that multiple season calving took place from the visual inspection of six δ^18^O sequences obtained on M1s. Due to the truncated sequences obtained on the M1s, it was not possible to model them and no comparison is possible with the results from the present study.

At Cheia, Borduşani, Bercy, Măgura and Alsónyék, birth seasonality data are also available for sheep (Fig. 4). Sheep are distinctive seasonal breeders. Under temperate latitudes, ewes experience alternating periods of fertility and infertility, defining a period in the year when most ewes in a herd are not sexually active^9^. A restricted breeding season for sheep has been clearly demonstrated in prehistoric Europe, with lambing occurring over a three- to four-month period^50^. A comparison of cattle and sheep birth seasonality shows that calving and lambing periods are of similar duration (Fig. 4; the calving period is longer at Măgura, although data may be biased by very small sample size), additionally arguing in favour of seasonal calving.

In the Marais Vernier nature reserve (France), where Highland cattle are kept alongside Shetland sheep, the latter give birth in March and April while the former do not give birth until May to July, when vegetation is more developed^51^. Unfortunately, the available data do not enable direct comparisons of the timing of births in cattle and sheep in archaeological contexts. The position of δ^18^O_max_ in the tooth crown not only depends on the date of birth, but also varies among species depending on the timing of tooth formation (the age at which crown formation is completed) and the delay in enamel mineralization. Not only are both parameters different in sheep and cattle, but they are also subject to considerable uncertainty^47^. For this reason, in sheep, the timing of births is determined by comparisons with modern references^47,50^. Such modern references are not yet available for cattle and future work is planned to focus on this point. A comparison of the timing and duration of the birth period in cattle and sheep would indeed allow for discussions on the complementarity of both species in terms of the availability of animal products throughout the year and the organization of pastoral tasks.

### The reasons for a restricted calving period

Different hypotheses may explain cattle births over a restricted period of the year:

1. *A physiological incapacity for year-round breeding*, i.e., seasonal anoestrus. If we assume that aurochs had true seasonal anoestrus, then it is possible that this characteristic had not yet disappeared in early cattle populations. The occurrence of a relatively high number of births outside the main calving period in LBK sites (9 out of 38 or 24% of births; Fig. 3C) does not support this hypothesis.
2. Inversely, *a false seasonal calving pattern* resulting from year-round breeding combined with higher calf mortality outside the optimal birth season. This hypothesis was not supported at Cheia (Supplementary Data S1), however, it cannot be ruled out at other sites. Autumnal calving was rarely observed in remnant populations of aurochs in Poland and those autumn calves were too weak to survive harsh winter conditions^12^. However, it is unlikely that infant mortality alone would create the observed seasonal pattern. In Chillingham feral cattle, breeding throughout the year, high infant mortality does not result in as strict a seasonal signal as the one observed in the prehistoric dataset^14^.
3. *Reproduction behaviour tuned to the availability of forage*, not necessarily involving a true physiological anoestrus but determined by vegetation dynamics and caused by seasonal fluctuations in the quality or quantity of forage resources, as is observed today in feral cattle populations (Supplementary Table S1).
4. *Intentional management* by herders, aiming to concentrate births in the optimal period of the year in terms of the availability of grazing resources. This breeding strategy would require careful management of relationships between females and males and would therefore necessitate a good understanding of the female reproductive cycle. It would also entail keeping males separately for part of the year. The occurrence of out-of-season births does not support strong control of the timing of breeding at LBK sites. However, different strategies may have been used in different periods and regions.

Hypotheses 3 and 4 would be equally possible and cannot be disentangled from the available evidence. Both hypotheses would suggest that these prehistoric agropastoral systems were still strongly constrained by environmental factors. At most considered locations in Europe, a potential factor restricting seasonal access to pastures would be snow cover in winter. The use of tree leaves as an alternative fodder, readily available in the Neolithic forested landscapes of Europe, has been demonstrated from archaeobotanical evidence^52^ and stable carbon isotope analysis on cattle remains: at Bischoffsheim^21,53^, Bercy^15^, Chotěbudice^18^ and Măgura^16^, where forest resources contributed to the cattle diet in winter, although to varying extents. Sheep winter foddering using forest resources is strongly suspected to have supported sheep lambing de-seasoning in the early Neolithic of southern France^6^. In contrast, despite evidence for supplementary foddering practices, in this present study we observe that cattle nutrition may not have been maintained throughout the year at a sufficient level to allow year-round reproduction. Year-round cattle breeding was observed in later periods, at the Early Bronze Age (around 2000 BC) sites of Irthlingborough and Gayhurst in Central England^3^ and at the Iron Age (first millennium AD) site of Pool in Orkney^4^. The technical, environmental or biological reasons for this change remain to be determined.

### A higher occurrence of out-of-season births at LBK sites

The higher occurrence of calves born out of the main birth season and which survived to adulthood at LBK sites may suggest weaker environmental constraints, potentially linked to favourable climate conditions. All sites included in this study were occupied during the Holocene climatic optimum (9000–5000 years ago), when a thermal maximum was attained in Europe^54,55^. Pollen-based reconstructions have shown that summer and winter temperatures in Central Europe reached a peak approximately 7000 years ago (± 500 years;^54^). The formation and the expansion of the LBK (5500–4900 cal BC) occurred during a period of winter warming and increased annual precipitation, bracketed by two rapid climatic events that were colder and drier^56^. In that respect, LBK farming systems may have experienced the warmest winters of the preindustrial Holocene and reduced temperature seasonality, at a time when the growing season also increased (estimated from growing degree-days above 5 °C;^54^). In these LBK contexts, the higher occurrence of out-of-season calving could similarly suggest favourable climate conditions, good quality forage available over a longer period throughout the year leading to less seasonally restricted breeding behaviour, as well as higher chances of survival for calves and lactating females. By contrast, the earliest occupations in the early Neolithic in the Balkans (the Starčevo-Çris-Körös complex) would have been partly contemporaneous with the ca 8.2 ky BP (~ 6200 cal BC) climate anomaly, when a marked decline in winter temperatures occurred in conjunction with drier conditions, over approximately 300 years^57,58^. The resulting stronger seasonality would have compromised year-round breeding.

As argued above, even though the environmental framework may create the conditions for year-round breeding, the calving pattern is ultimately determined by herding practices. Intentional management aiming to extend the calving season, in order to produce milk over a longer period of the year, is difficult to demonstrate for the LBK. Nevertheless, if not intentionally scheduled, out-of-season births were at least not actively prevented. Implications for the reconstruction of LBK agropastoral systems are twofold. First, unintentional out-of-season calving would suggest that males and females in LBK herds were herded together all year round and could be the sign of little control on cattle breeding. Second, calving occurring mainly over a restricted period of the year, along with a significant proportion of out-of-season births, can be introduced into models aiming at reconstructing LBK farming systems. Saqalli et al.^59^, considering the cultivated crops and livestock species identified at LBK sites, have proposed a calendar of agropastoral activities in light of agronomic and zootechnical means available to preindustrial farming. Following them, calving took place in the spring, at a time when intense labour was also required in cultivated fields for sowing and weeding. Although the birthing season cannot be inferred from our results, out-of-season births seem to occur six months apart from the main calving season (Fig. 3). If the main calving peak took place in the spring, out-of-season births occurred in autumn. While relieving pressure on the spring tasks, autumn calving would, on the other hand, happen while labour was mobilised for the sowing of winter cereals (wheat)^59^. The impact of overlapping activities for the sustainability of these farming systems would depend on the size of fields and herds, and the capacity to mobilize labour, whether this was managed at the household or the settlement scale^59^.

### Consequences for milk availability and cheese-making

The presence of dairy lipid residues in ceramic pots provides direct evidence of milk processing in the sixth millennium BC in the Balkans^29,31^, where they were in some instances directly dated^31^, as well as in the Carpathian Basin^24^, southern France^28^, eastern France^60^ and in Poland^27,61,62^. In the early Neolithic contexts in Central Europe, cattle predominate in the faunal assemblages and demographic management inferred from mortality profiles strongly suggests milk exploitation^30^. Such evidence is also available in the Hamangia and Gumelniţa cultures in Romania^33,36^, as well as in the Chasséen in France^37^. The restriction of the main calving period to a period of two to four months would result in fluctuating milk availability and complete absence of cattle milk for some periods of the year. Such intervals would have been further extended by the presumably shorter lactation and lower milk yields of prehistoric cattle (6–8 months in unimproved modern European cattle breeds:^63–65^; including significantly reduced milk production in the late stage of lactation). Moreover, conception intervals are dependent on the nutritional plane of the mother and in unmanaged herds calving intervals can last for over a year^14,66,67^. Consequently, early cattle may have calved every other year and the level of milk production could have fluctuated both throughout the year and from year to year.

Fluctuating milk availability could have created the need to transform it into a storable product. Milk was likely processed already in the earliest stages of the history of dairying, in line with widespread lactose intolerance among adults^68,69^, but also simply for preservation. Fresh milk that cannot be consumed within a few hours has to be handled to avoid microbial growth. Products with a semi-solid texture, such as sour milk and soft cheese, obtained by lactic acid fermentation, are storable for several days. To compensate for seasonal interruptions in animal lactation, however, milk has to be transformed into products with a shelf life bridging several seasons, like hard cheeses. Ethnographic literature provides ample evidence for the practice of producing very hard dry cheeses in pastoral societies, where milk is a seasonal food^70–73^. Sedentary communities also produce cheeses with a long shelf life. At Kizilkaya in Central Anatolia, for example, each family prepares c. 15–20 kg of cheese, mostly made from sheep's milk, or a mixture of sheep's and cows’ milk, for storage and consumption during winter^74^. Ceramic sieves, used for separating curds from whey, attest to cheese manufacture in sites from different cultural complexes in the Early and Middle Neolithic in Europe^26,75,76^. Other containers made of perishable materials, such as bark and grass, may also have been used as strainers, as shown by specimens recovered later in the Bronze Age^75^. Therefore, cheese-making enabled Neolithic populations in Europe to overcome, and was probably a direct consequence of, seasonal cattle breeding and the resulting seasonally fluctuating milk availability. This does not preclude the practice of cheese making earlier in the history of dairy exploitation in the Near East, under different environmental constraints and possibly for other reasons. Since prehistoric times, even though cattle milk has become available throughout the year, hard cheeses are still being processed but appreciated primarily for their taste.

## Conclusions

The available data suggest that seasonal calving prevailed in the sixth to fourth millennia cal BC in Europe. It is not possible for now to determine whether this pattern reflects cattle breeding behaviour in tune with the seasonal vegetation cycle or whether it is also partly the result of intentional management by herders. In both cases, seasonal calving would mean that cattle agropastoral systems during this period were still strongly constrained by environmental factors, in particular grazing resources, despite the attested use in some regions of forest leafy resources in winter. The higher occurrence of out-of-season births in LBK contexts, at a time of winter warming and increased annual precipitation in Central Europe, would support the hypothesis that climate and availability of forage played a determinant role in shaping the seasonality of cattle breeding.

A consequence of seasonal calving in prehistoric Europe would have been an irregular supply of cattle milk throughout the year and from year to year. In these pastoral systems, sheep lambing occurred from late winter to early summer^50^. It is likely that calving would also have been centred on the spring, based on observations of feral cattle populations from European nature reserves. However, the precise timing of cattle and sheep birth periods, and the possible time lag between them, would need to be determined before one can address the complementarity of cattle and ovicaprids exploitation. In cases where sheep and cattle milk were jointly exploited, this could have slightly extended the period of milk availability. Nevertheless, there would still have been periods of milk deprivation, which could have been overcome by making hard cheese, to be stored over several seasons for delayed consumption. In addition to supervising calving, tending calves in their first weeks and milking, making hard cheese, which is a multiple stage and demanding task^74^, would also have greatly constrained the agropastoral calendar, shaping farmers’ lives. The converse solution of extending the birth season and stretching fresh milk consumption over a longer period of the year would have involved intense foddering throughout the winter. Depending on the number of animals to be fed, the length of the foddering period, the type of fodder (grass/tree leaves) and the distance at which fodder was harvested^77^, this could have required intense investment. Therefore, the labour and organization resulting from seasonal calving, as observed in this study, is difficult to compare with systems where year-round calving is sustained.

Our study opens the way to diachronic research to define when year-round breeding became more prevalent in Europe, whether this was triggered by changes in society that demanded year-round sources of milk, and how ancient agropastoral societies became capable of maintaining aseasonal breeding.

### Material and methods

All teeth included in the study are archaeological material. All samples were taken from M3s (105 M3s in total). This choice was dictated (1) by the necessity to secure tooth identification in assemblages sometimes mostly composed of isolated teeth, where second and first molars are difficult to distinguish; (2) and the fact that a higher number of teeth with light wear (i.e., preserved crown height) could be sampled. To avoid potential misidentification with the aurochs, which is very similar in morphology to Neolithic cattle, the domestic status of the selected teeth was assessed using morphometric criteria (Supplementary Data S2). Most of the M3 are lower molars except at Măgura^16^ and Alsónyék (this study) where a higher number of upper M3 were available. Left and right molars have been used; their belonging to different individuals was assessed on the basis of tooth size and wear. The Cheia assemblage additionally provided 11 lower dP4 and seven lower M1 belonging to 15 calves.

The δ^18^O values were measured on the carbonate fraction of enamel bioapatite. Enamel was sequentially sampled following the procedure described in^46^ on the anterior lobe of the M3 and M1, and on the posterior lobe of the dP4 (except for CHE Bos1dP4 sampled on the anterior lobe; and CHE Bos4 dP4 sampled on the middle lobe). Enamel powders were pre-treated to eliminate diagenetic carbonates (0.1 M acetic acid for 4 h at room temperature, 0.1 ml/mg). Pre-treated enamel samples weighing ~ 600 μg were reacted with 100% phosphoric acid at 70 °C in individual vessels in an automated cryogenic distillation system (Kiel IV device), interfaced with a DeltaVAdvantage isotope ratio mass spectrometer at the “SSMIM” IRMS service (MNHN, Paris). The analytical precision for each run, estimated from 6 to 8 analyses of our laboratory carbonate standard (Marbre LM, expected value − 1.83‰ calibrated to the NBS 19 international standard) was always lower than 0.05 ‰.

The δ^18^O sequences measured in the M3 were modelled using an equation derived from a cosine function (^47^; see Supplementary Method S1) in order to define the position (distance to enamel-root junction) of the maximum value (x_0_). The period of the cycle (X, distance over which the isotopic record covers one annual cycle) was used to normalize the distances (x_0_/X) in order to eliminate inter-individual variability in tooth size. This normalization procedure does not eliminate variability in the timing of tooth growth. Although the chronology of tooth development in cattle was found to be remarkably similar in different dairy and beef breeds^48^, variability exists between individuals. Mean standard deviations of 21 days and 42 days are given for the different development stages of the first and second molars respectively in varied cattle breeds and nutritional systems^78^. The variability of the third molar might be even greater^79^, but has not been clearly defined.

The x_0_/X ratio defined for each modelled δ^18^O sequence may be directly compared between specimens. The spread of these ratios within a population reflects the duration of the period of birth. Circular charts are used to reflect the cyclical nature of seasonality (^50^; see Supplementary Method S1): when (x_0_/X) reaches 1, it also reaches 0. Student statistics were applied to the small size samples. Births occurring outside the 80% confidence interval on the site scale (or 95% on the European scale), were considered as outliers. The 68% confidence interval around the mean (excluding outliers) defines the main period of births. Results from these calculations are given in Supplementary Tables S15 and S16. Histograms showing the distributions of cattle births when combining data on a European scale are shown in Supplementary Fig. S12. They all resemble normal distribution. The data for site or group of sites are assumed to be from normal distributions as they are sub-samples of the combined datasets. The four Early Neolithic sites in Serbia, which yielded a small number of modelled δ^18^O sequences (Starčevo-Grad = 1; Magareći mlin = 3; Divostin = 1; Blagotin = 4) were considered together for data treatment. The same was applied to the LBK sites in Czech Republic. Černý Vůl (N = 1) and Těšetice-Kyjovice (N = 1) were grouped with Chotěbudice (N = 10), while Hârşova (N = 1) was grouped with Borduşani (N = 9).

The comparability of results obtained on the upper and lower M3 was investigated at Borduşani, where the average x_0_/X ratio obtained from 10 lower M3 was compared to the average x_0_/X ratio obtained from 4 upper M3 (see Supplementary Method S2). The highlighted shift (+ 0.43 in upper M3, or 5.2 months) is considerable and precludes a direct comparison of data obtained from lower and upper M3. A − 0.43 correction was applied to the Măgura and Alsónyék datasets before comparison with other sites. This correction, acquired on a limited number of individuals, must be refined in the future and for this reason datasets mixing upper and lower teeth on a site scale were not included in this study, as the mixing would impact the estimation of the duration of the calving season.

## Supplementary Information

Supplementary Information
