# Supplementary material for: Seasonal calving in European Prehistoric cattle and its impacts on milk availability and cheese-making
Source: Sci Rep. 2021 Apr 14;11:8185. doi: 10.1038/s41598-021-87674-1 (PMC8046818; doi:10.1038/s41598-021-87674-1)
Supplement: Supplementary file 1 — Supplementary Information [file 41598_2021_87674_MOESM1_ESM.pdf]

Supplementary Information

Title: Seasonal calving in European Prehistoric cattle and its impacts on milk availability and cheese-making.

Authors: M. Balasse, R. Gillis, I. Živaljević, R. Berthon, L. Kovačiková, D. Fiorillo, R.-M. Arbogast, A. Bălăşescu, S. Bréhard, É. Á. Nyerges, V. Dimitrijević, E. Bánffy, L. Domboróczki, A. Marciniak, K. Oross, I. Vostrovská, M. Roffet-Salque, S. Stefanović, M. Ivanova.

List of Supplementary information (by order of appearance):

Supplementary Table S1: Seasonality of reproduction in unmanaged herds of feral cattle breeds kept in nature reserves in Europe.
Supplementary Table S2: List of the sites included in the study and dates.
Supplementary Note S1: Sites description
Supplementary Table S3 to S9: Results from the sequential analysis of enamel $\delta^{18}O$ values:
Table S3- Balatonszárszó-Kis-erdei-dűlő (Hungary).
Table S4- Apc (Hungary).
Table S5- Starčevo-Grad (LBSG), Magareći mlin (LBMM), Divostin (LBDI), Blagotin (BL) (Serbia).
Table S6-Těšetice-Kyjovice (Czech Republic).
Table S7- Borduşani (Romania).
Table S8- Auriac (France).
Table S9- Alsónyék (Hungary).
Supplementary Figures S1 to S7: Results from the sequential analysis of enamel $\delta^{18}O$ values:
Figure S1 - Balatonszárszó (Hungary).
Figure S2 - Apc (Hungary).
Figure S3 - Starčevo-Grad (LBSG), Magareći mlin (LBMM), Divostin (LBDI), Blagotin (BL) (Serbia)
Figure S4 - Těšetice-Kyjovice (Czech Republic).
Figure S5 - Borduşani (Romania).
Figure S6 - Auriac (France).
Figure S7 - Alsónyék (Hungary).
Supplementary Method S1: Modelling the $\delta^{18}O$ sequences: methodological steps
Supplementary Table S10: Results from the modelling the $\delta^{18}O$ sequences
Supplementary Data S1: Results from the stable isotope analysis of young cattle dP4 and M1 at Cheia (Romania).
Supplementary Figures S8: Results from the oxygen isotope analysis of the cattle dP4 and M1 at Cheia
Supplementary Table S11: Results from the sequential analysis of enamel $\delta^{18}O$ values: Cheia (Romania)
Supplementary Data S2: Osteometric assessment of the status of the teeth included in the study.
Supplementary Figures S9: Measurements taken on the *Bos* lower M3 at the early Neolithic sites
Supplementary Figures S10: Measurements taken on the *Bos* lower M3 at the Middle Neolithic sites in France and Chalcolithic sites in Romania
Supplementary Figures S11: Measurements taken on the *Bos* upper third molars (M3) from Borduşani, Măgura and Alsónyék, compared to the Durankulak dataset (Manhart 1988).
Supplementary Table S12: Measurements taken on the *Bos* lower M3.
Supplementary Table S13: Measurements taken on the lower M3 of large Bos specimens (aurochs?)
Supplementary Table S14: Measurements taken on the *Bos* upper M3s.
Supplementary Figure S12: Histograms showing the distribution of cattle births when combining data on a European scale. Results from Gaussian curve fitting.
Supplementary Table S15: Results from the calculation of the main calving peak (68% of births) at individual sites or on a regional scale.
Supplementary Table S16: Results from the calculation of the 95% and 68% confidence intervals for cattle births on the European scale.
Supplementary Method S2: Assessment of the comparability of results obtained on the upper and lower M3.

Supplementary Figures S12: Comparison of the results obtained from upper and lower M3 at Borduşani.
Supplementary Table S15: Comparison of the results obtained from paired lower and upper M3 at Borduşani (PBORD) and Hârşova (HVA).
Supplementary Figures S13: Correction of the results obtained on upper M3s at Măgura (MAG) and Alsónyék (ALS),

Supplementary Table S1:

Seasonality of reproduction in unmanaged herds of primitive cattle breeds kept in nature reserves in Europe.

Location	Breed	Calving period	Date	Number of observations	Ref
Donana nature reserve (southwestern Spain)	“primitive” breed	Feb-August 95% (Mar-May 58%)	1983-1989	113	(1)
Naturpark Rhein-Taunus (Germany)	Scottish Highland cattle	March and April (91%)	1978-1982	22	(2)
Marais Vernier reserve (northern France)	Scottish Highland cattle	May to July	1979-1985	18	(3)
Oostvordersplassen nature reserve (Netherlands)	Heck cattle	March to June (84%)	2003	120	(4)
Imbos. Veluwezoom National park (Netherlands)	Scottish Highland cattle	April to Sept. mainly April-June	1983-1987	14	(5)
Basque country (Spain)	Betizu cattle	March	unknown	unknown	(6)
Chillingham park (northern England)	White cattle	year-round breeding	1862-1898<br>1953-1984	362<br>264	(7)

References

(1) Lazo A., 1992. Caracteristicas poblacionales del ganado bovino asilvestrado de la Reserve Biologica de Donana (S.O. de Espana). *Acta vertebrata* 19 (1-2), 85-96.

(2) Reinhardt C., Reinhardt A., Reinhardt V., 1986. Social behaviour and reproduction performance in semi-wild Scottish Highland cattle. *Applied Animal Behaviour Science* 15, 125-136.

(3) Lecomte T., Le Neveu C., 1986. *Le Marais Vernier. Contribution à l'étude et à la gestion d'une zone humide*. Université de Rouen Haute-Normandie. Rouen.

(4) Van Vuure, pers. com. in Gidney 2013: Gidney L.J., 2013. *Offspring of the Aurochs: A comparison of a reference collection of Dexter cattle skeletons with archaeological and historical data*. Durham theses. Durham University. Available at Durham E-Theses Online: http://etheses.dur.ac.uk/10561/

(5) van Wieren S.E., 1988. Runderen in het bos. Begrazingsproef met Schotse Hooglandrunderen in het natuurgebied de Imbos [Cattle in the forest. A grazing experiment with Scottish Highland cattle in the nature reserve De Imbos]. Instituut voor Milieuvraagstukken. Amsterdam.

(6) Gómez M., Plazaola J.M., Seiliez J.P., 1997. The Betizu Cattle of the Basque country. *Animal Genetic Resources Information* 22, 1-5.

(7) Hall S.J.G., Hall J.G. ,1988. Inbreeding and population dynamics of the Chillingham wild cattle (*Bos taurus*). *Journal of Zoology* 216, 479-493.

Supplementary Table S2: List of the sites included in the study and dates.

Site	Chronocultural complex	Dates cal BC	N (tooth)	Stable isotope data
Divostin	Starčevo	6350-5850 (1,2)	1 M3	This study
Blagotin	Starčevo	6250-5900 (2,3)	4 M3	This study
Magareći mlin	Starčevo	6200-5700 (2,4,5)	3 M3	This study
Măgura-*Boldul lui Moş Ivănuş*	Starčevo-Criş I	6050-5750 (6)	4 M3	(6)
Starčevo-Grad	Starčevo	6000-5300 (2,3,7)	1 M3	This study
Alsónyék-Bátaszék	Starčevo	5800-5505 (8)	11 M3	This study
Apc	LBK	5470-4950 (9)	7 M3	This study
Balatonszárszó-Kis-erdei-dűlő	LBK	5350-4900 (10)	5 M3	This study
Ludwinowo	LBK	5300-5000 (11)	7 M3	(22)
Těšetice-Kyjovice	LBK	5300-5000 (12)	1 M3	This study
Chotěbudice	LBK	5400-5100 (13, 14)	10 M3	(23)
Bischoffsheim	LBK	5300 to 4850 (15)	7 M3	(24)
Černý Vůl	LBK	5200-5000 (16)	1 M3	(23)
Cheia	Hamangia	5215-4855 (17)	12 M3, 11 dP4, 7 M1	(25) and this study
Borduşani-*Popină*	Gumelniţa A2	4490-4263 (18)	13 M3	(24) and this study
Hârşova-tell	Gumelniţa A2	4340-4035 (19)	1 M3	This study
Bercy	Chasséen	c. 3900 (20)	9 M3	(26)
Auriac	Chasséen	3986-3799 (21)	8 M3	This study

LBK = Linearbandkeramik; 1: McPherron et al. (1988) ; 2: Porčić et al. (2020, 2021); 3: Whittle et al. (2002); 4: Tasić (1993); 5: Pinhasi et al. (2005); 6: Balasse et al. (2013); 7: Stefanović et al. (2019); 8: Oross et al. (2013); 9: Domboróczki et al. (2016); 10: Oross et al. (2020); 11: Nowak et al. (2017); 12: Vostrovská (2018); 13: Rada (1981); 14: Šumberová (1991); 15: Denaire et al. (2017); 16: Řídký et al. (2008); 17: Balasse et al. (2014); 18: Gillis et al. (2013); 19: Bem (2001); 20: Lanchon et al., 2000; 21: Vaquer & Gandelin (2018); 22: Kendall et al. (2019); 23: Berthon et al. (2018); 24: Gillis et al. (in press); 25: Balasse et al. (2014); 26: Balasse et al. (2012a).

Supplementary Note S1: Sites description

Divostin (Serbia, Starčevo, 6350-5850 cal BC) is located in the Šumadija region in Central Serbia. It is one of the earliest Starčevo sites in the region (Porčić *et al.*, 2020, 2021). The Early Neolithic phase of occupancy is manifested by semisubterranean and ground level buildings, fireplaces, hearths and ovens, pits, coarse, fine and painted pottery, figurines, chipped, polished and osseous tools, animal and plant remains, and several burials (McPherron and Srejović, 1988). The faunal sample (NISP=2,401) was dominated by the remains of domestic ruminants - cattle (46.5%) and ovicaprids (40.9%). In comparison, the remains of pig (3.5%), dog (0.7%), and wild animals (8.3%) were fewer in number. Mortality profiles were constructed on the basis of stages of epiphyseal fusion. In the cattle population (MNI=98), adult individuals predominate with more than 60%, followed by subadults. The remains of young animals (before their first winter) were scarce, however their exact number was not specified. Sheep and goat were mainly slaughtered as lambs or subadults, but the latter are also represented by adult, mature and senile animals (Bökönyi, 1988). Among the small sample of lipid-bearing pottery sherds (3 out of the 43 analyzed), ruminant dairy fats were detected in one sherd (33%), and non-ruminant adipose fats in two sherds (67%; Ethier *et al.*, 2017).

Blagotin (Serbia, Starčevo, 6250-5900 cal BC) is located in the hilly and previously densely forested region of Šumadija in Central Serbia. It is one of the earliest Starčevo sites in the region (Whittle *et al.*, 2002; Porčić *et al.*, 2020, 2021). The settlement was fairly small, with one large central semisubterranean building encircled with smaller pit-buildings. Several rubbish pits were also

discovered, as well as clay altars, figurines and grain models, monochrome and painted pottery, chipped and polished stone tools, bone tools, numerous animal bones, plant remains, and one human burial (Stanković and Greenfield, 1992; Stanković and Leković, 1993; Greenfield, 2000; Greenfield and Jongsma, 2006; Greenfield *et al.*, 2014). In the large faunal sample (NISP=32,037), ovicaprids were most numerous (59.4%), followed by cattle (30.8%), whereas the remains of pig (1.3%) and dog (0.2%) were few. Wild animals were represented with 6.9%. Mortality profiles of sheep and cattle (mainly represented by subadult individuals, followed by adults and juveniles) suggest a primary products exploitation, while the culling pattern of goat (mainly represented by adult individuals, followed by subadults) indicates a mixed meat and milk exploitation. In cattle, foetal/infantile individuals represented 0.9%, and juvenile 25% of aged remains (N=1,758). However, their age distributions could not be associated with narrow monthly ranges (Greenfield *et al.*, 2014). Lipid residue analyses of pottery have confirmed the presence of ruminant dairy fats (n=7; i.e. 78% of lipid-bearing sherds) and ruminant adipose fats (n=2; i.e. 22% of lipid bearing sherds), indicative of both dairying and carcass product processing (Ethier *et al.*, 2017).

Magarići mlin (Serbia, Starčevo, 6200-5700 cal BC) is situated on an alluvial plain formed by the meandering of the Danube, in the lowland landscape of the Vojvodina province in northern Serbia (southern part of the Carpathian Basin). It is one of the earliest Starčevo sites in Vojvodina (Porčić *et al.*, 2020, 2021). The remnants of the Neolithic settlement included several semisubterranean dwellings and associated rubbish pits, monochrome and white painted pottery, polished and chipped stone artefacts, animal bones and mollusc shells (Leković, 1988). The mammal faunal sample (NISP=603) mainly comprised of bones of domestic ruminants – cattle (60.1% of all remains identified to the level of species/genus) and ovicaprids (26.1%), whereas domestic pig and wild animals were fewer in number (1.1% and 9.0%, respectively; Stojanovski *et al.*, 2020). In terms of cattle mortality profiles, constructed using lower teeth and mandibles (MNI=15), all age stages were present, with a similar number of young and adult individuals. Young animals were represented by 3 (20%) individuals aged 0-6 months, and 1 (6.7%) individual aged 6-12 months. Isotopic intra-tooth variations in two calf individuals demonstrate that they were culled well after weaning occurred (Stojanovski *et al.*, 2020). Ruminant dairy fats were detected in 62% lipid-bearing sherds (n=8), whereas the organic residues from 31% lipid-bearing sherds (n=4) are indicative of ruminant carcass product processing (Stojanovski *et al.*, 2020).

Măgura-*Boldul lui Moş Ivănuş* (Romania, Starčevo-Criş I, 6050-5750 cal BC) is located in the Teleorman river floodplain in southern Romania. The settlement was occupied by communities from the Starčevo-Criş I culture, closely related to the earliest Neolithic in the Balkans (Andreescu and Mirea, 2008). The faunal remains testify of the major role played by husbandry in subsistence, as evidenced by the overwhelming representation of domestic animals among mammal remains (92% of identified remains, NISP=4,390): caprines, of which sheep greatly outnumbered goats, were strongly represented (60%), together with cattle (31%) and a few domestic pigs. The cattle and caprines mortality profiles suggest a primary orientation towards meat production, although caprines may also have been exploited for milk (Bălăşescu, 2014). Hunting, focusing primarily on red deer, wild boar and aurochs, played a secondary role in the economy. The ecological preferences of the wild mammals identified at the site indicate the co-existence of forested and open areas, while a rich water environment was also exploited. Cereal cultivation is attested indirectly (tools, charred cereal grains in ceramic pastes, grain impressions on pottery). A stable isotope study concluded a similar habitat and trophic level for both the domestic stock and wild herbivores, suggesting extensive herding in open areas. The contribution of forest resources fodder to the winter diet of sheep and cattle was suspected in some instances (Balasse *et al.*, 2013).

Starčevo-Grad (Serbia, Starčevo, 6000-5300 cal BC), the eponymous site of the Starčevo culture, is located on the Danube bank in the province of Vojvodina in Serbia, in the southernmost part of the Carpathian Basin. Based on radiocarbon dates (Whittle *et al.*, 2002; Porčić *et al.*, 2021), it was

determined that it was settled during the later phase of the Starčevo culture. Several excavation campaigns uncovered the evidence of numerous semisubterranean features and pits, painted pottery, stone and bone artefacts, as well as several burials, and animal bones (Grbić, 1930; Fewkes *et al*., 1933; Ehrich 1977; Živković *et al*., 2011). The faunal sample from secure Neolithic contexts comprised 1,450 specimens, with the majority originating from domestic ruminants – cattle (43.9%) and ovicaprids (20.2%). Wild animals were represented in fairly significant numbers (23.2%), whereas domestic pig (2.8%) and dog (0.5%) remains were few (Clason, 1980). Cattle mortality profiles were constructed using lower teeth and mandibles (MNI=14). The number of young individuals significantly exceeded the number of adults, with 4 (28.6%) animals aged 0-6 months, and 1 (7.1%) animal aged 6-12 months. Sequential stable isotope analysis of dentine collagen of one calf individual indicates that it was still suckling at the time of culling (Stojanovski *et al*., 2020). Ruminant dairy fats were detected in 48% lipid-bearing pottery sherds (n=12). In addition, 40% (n=10) and 8% (n=2) lipid-bearing sherds yielded evidence of carcass product and plant processing, respectively. Furthermore, compound-specific radiocarbon analyses (CSRA) of dairy lipids were also performed, providing the first direct dating (6860±46 BP; i.e. 5845-5650 cal BC) of the occurrence of milk products in the region (Stojanovski *et al*., 2020). The biomolecular analysis of human dental calculus showed that none of the analysed individuals (n=3) display confident proteomic evidence for milk consumption, which suggests that it was not consumed in its raw form, but as dairy products (Stojanovski *et al*., 2020). The appearance of new foodstuffs (baby gruels) was also suggested based on traces of use on bone spoons, corresponding to human milk teeth marks (Stefanović *et al*., 2019).

Alsónyék-Bátaszék (Hungary, Starčevo, 5800-5505 cal BC) is situated in southern Hungary on the right bank of the Danube. It was first occupied by Early Neolithic people from the Starčevo culture, and later by Neolithic communities from the Linearbandkeramik (LBK), the Sopot and the Lengyel culture. The Early Neolithic features include clay ovens and graves, but the vast majority are pits, some of which may have been related to clay extraction, which eventually got filled with refuse. The filling of these pits contained more than two tons of burnt daub from house walls, abundant pottery, animal bones and shells (Bánffy *et al.* 2010; Bánffy et al 2016, Bánffy and Höhler-Brockmann 2020). The animal remains (NISP=8,997) included a low proportion of wild game (11 %). Cattle (43%) and caprines (43%) dominated the domestic species (89 %). Pigs were also attested, although their contribution to the assemblage was minimal (2 %; Nyerges and Biller, 2015 and Biller forthcoming).

Apc-Berekalja (Hungary, LBK, 5470-4950 cal BC) can be found in Northern Hungary some 70 kms to the northeast of Budapest. The site was occupied from the Neolithic through the Copper and Bronze Age up until the Migration Period. The archaeological area covers more than 30 ha out of which almost 2 ha was excavated. On the excavated surface in 2009 and 2014 the Neolithic LBK settlement remains seemed to be the most significant (Domboróczki et al., 2016). Approximately 30 post-framed LBK houses were identified here belonging to various types (Domboróczki, 2018). Among the houses, pits and graves were situated. The collected LBK material consisted mostly of pottery and bone. From the total of 4,169 animal remains found at the site, the largest part (2,254 fragments) came from the Neolithic period, from which 2,232 could be identified at least on the level of biological order. Of these, 95.6 ‰ (2,131 fragments) originate from domestic mammals: cattle (1,578 fragments, 70.6 % of the identified remains), sheep and goats (385, 17.3 %), domestic pig (163, 7.3 %) and dog (5, 0.2 %). The proportion of wild animal remains is relatively low: only 95 fragments (4.3 %) form this group. Age-at-death analysis of of sheep remains suggested that animals were slaughtered for meat while goats may have been managed for milk (Gillis et al., 2019). Similar analysis on cattle remains indicate that animals were managed for both milk and meat (Gillis et al., 2017). In cattle, the 0-6 m and 6-15 m age classes include respectively 4.5 % and 11 % of dental remains (N = 67; Gillis et al., 2017).

Balatonszárszó-Kis-erdei-dűlő (Hungary, early and developed LBK, 5350-4900 cal BC) is situated to the west of the Danube, on a low plateau close to the southern shore of Lake Balaton. Discovered during rescue excavations conducted before the construction of the M7 motorway. The investigated area of

the Neolithic settlement totals to around 10 ha. The unparallel excavations revealed features relating to 48 one-time house plans associated with the LBK (Bánffy and Oross 2009, 2010), traces of another 15 buildings were identified in course of the assessment. The settlement consists of two major parts with five different pottery style groups revealed at the site. Moreover, 43 settlement burials were also recovered. The teeth analysed here come from features No B.3627 (Bos1), B.3627 (Bos 2), B.327 (Bos 3), B.4327 (Bos 4) and B.4297 (Bos 5).

Ludwinowo 7 (Poland, LBK, 5300-5000 cal BC). The LBK settlement of Ludwinowo 7 lies on the edge of a small plateau overlooking the Vistula valley in the Kuyavia region of the central Polish lowlands. Excavated in 2000-1 and 2008-9 ahead of motorway construction, features uncovered included postholes, long pits (*Längsgruben*) and other pits. These features were interpreted as the remains of approximately 26 'households' including 14 longhouses. The initial occupation of the site began in the late 6th millennium BC (Kuyavian phase I, corresponding to late *Älteste* LBK). However, the main period of occupation spans approximately 250 years, from c. 5250 to 5000 BC (Kuyavian phases IIA to III, or early *Notenkopf* to *Jüngere* LBK; Pyzel *et al.* 2019, Pyzel 2019). Extensive sampling for organic residue analysis was carried out on 519 sherds from four different pot types excavated across the site and dated from phases I to III (Salque et al. 2013, Roffet-Salque et al. 2019). The lipid extracts from the perforated sherds (originally interpreted as sieves; Bogucki 1984) were dominated by ruminant dairy fats, strongly suggesting they had been used in cheese production (Salque et al. 2013). The globular pots (or *Kümpfe*) yielded residues dominated by ruminant adipose fats or mixtures of ruminant and non-ruminant adipose fats (81/116 or 70%) or ruminant dairy fats (35/116 or 30%; Roffet-Salque et al. 2019). Several collared flasks showed traces of beeswax, possibly used as a sealant (Salque et al. 2013, Roffet-Salque *et al*. 2015, Roffet-Salque et al. 2019). The faunal assemblage consisted of mainly bones from domesticated animals (95.8%) of which 72.5% were cattle (Osypińska and Abłamowicz 2019). Sheep and goat only represented 12.1% of the assemblage, predominantly coming from animals aged 2-4 years old. Age-at-death analysis from cattle teeth, from Phase IIb and III, indicated that they were managed for milk and meat (Gillis et al. 2017). In phase IIb (N = 40), the 0-6 m age class includes 4.7 % of cattle dental remains and the 6-15 m age class includes 11 %; the same figures for phase III (N = 83) are 10.5 % (0-6 m) and 12,8 % (6-15 m) (Gillis et al., 2017). Cattle remains from juvenile and young animals with evidence of marrow exploitation dominate clay pits and have been interpreted as evidence of large-scale community feasting events (Marciniak 2005; Johnson et al, 2021).

Těšetice-Kyjovice (Czech Republic, LBK, 5300-5000 cal BC). The site is situated in the foreland of the Bohemian-Moravian Highlands, on the boundary line between the Pannonian and Hercynian biogeographical subregions. The prehistoric settlement area spreads out on the south-eastern plateau at an elevation of 265–290 m, cut by the narrow, deep valley of the Únanovka stream. Systematic excavation at Těšetice-Kyjovice since 1967 has uncovered a multi-period site with settlement remains from the Neolithic Period, Bronze Age and Iron Age. The LBK settlement was excavated on an area of 2.4 hectares; at least 22 badly preserved outlines of timber post longhouses, 11 inhumation graves and more than 123 settlement features were found. A geophysical survey on an area of 11.1 hectares ascertained that the major part of the LBK settlement area extends further northeast of that and at least 55 other longhouses with longitudinal pits arranged in several rows can be identified there (Vostrovská, 2018). Cattle were the most dominant species at 45%, with caprines (both sheep and goat) following at 30% and pigs at ~20% (Dreslerová, 2006). Domestic dogs were also very occasionally identified. Of the wild species represented aurochsen were the most common, followed by, red deer, roe deer, and wild boar. Other wild species of rodent and bird were also identified in small numbers. For cattle, there was some young slaughter before 1 year (4.8 % of dental remains belong to the 0-6m age class, and 10 % to the 6-15 m age class; N = 27, Gillis et al., 2017), but the main slaughter event was between 1.5 and 3 years, with no further kill off before 4 years. The young slaughter could represent post lactation slaughter, followed by meat age slaughter, with older animals being kept on for milk production. It is likely that bone marrow was processed on an intermediate scale at Těšetice. The FFI score and fracture history profiles for the comparable contexts suggest that processing marrow

was not standardised across the site. It is possible that bones may have been boiled or roasted before fracture from the presence of slice marks and roasting on bones (Johnson et al., 2018).

Chotěbudice (Czech Republic, LBK, 5400-5100 cal BC) has yielded nineteen ground plans of long houses and 800 LBK structures spanning a long-term occupation ranging from LBK stages IIa to LBK IIIb (Rada, 1981; Šumberová, 1991). Domestic cattle occur as the main species in the archaeozoological assemblage throughout the LBK occupation (total LBK NISP=4,788). Cattle represent from 63% to 85% (depending on the occupation phase), followed by sheep, goat and pig. Cattle kill-off patterns for the three most representative LBK phases show similar trends over a period of roughly 200 years and suggest a dual exploitation of milk and meat (Kovačiková et al., 2012). Depending on the occupation phase, the relative proportion of cattle in the 0-6 m and 6-15 m age classes respectively varies from 2.1 % to 19.6 % and from 8.7 % to 18.3 % (N varies from 21 to 126; Gillis et al., 2017).

Bischoffsheim (France, LBK, 5300-4850 cal BC). The village of Bischoffsheim is located about twenty kilometers west of Strasbourg, on a vast loess veneer extending at the foot of the sub-Vosgian hills. The excavation campaign carried out in 2002 resulted in the discovery of nearly 2000 structures, the majority of which belonged to 40 identifiable houses, spread over approximately 3 hectares. Only the eastern limit of the site was reached; we know from previous discoveries that the ribboned habitat extends westward over an area probably as large as that of the studied area. The analysis of the decorated ceramics from the houses' construction pits allows revealed six stylistic stages covering the ancient, middle and recent stages of the Rubané culture. Of the 40 observed buildings, 32 exhibit readable plans linking them with varying degrees of certainty to one of the three main types defined by P. J.-R. Moddermann: 22 belong to the large tripartite house category, composed of a front part with an attic, a central part and a rear part often materialized by a foundation ditch; seven can be assimilated to bipartite houses (without attic) and only three to small houses. The unearthed archaeological remains form one of the richest collections in Lower Alsace for the Early Neolithic period. The lithic industry meets what one would expect from a Lower Alsace site and the corpus of bone tools is the most important in Alsace after that of Rosheim "Saint-Odile". The site is unique due to the frequency of milling equipment, a feature peculiar to foothill sites, and to the abundance of ochre dye, which was exploited on a nearby hill. The scarcity of ceramic imports from other provinces of the Rubané, the relative poverty of the unearthed artefacts when compared to those of the neighboring site of Rosheim "Sainte-Odile" lead us, as it stands, to consider the habitat of Bischoffsheim as a satellite site which depends on the central site of Rosheim. Cattle and pigs were the predominately managed domesticates followed sheep/goat. Cattle appeared to be managed for both milk and meat, with the latter becoming more predominant during towards the end of the Rubané (Gillis et al. 2017). Organic residue analyses carried out on 290 sherds spanning from the Early LBK IIB to the Late LBK IVB showed a rather weak evidence for the use of milk at the site. Of the 67 sherds bearing archaeological animal fats, 0.5% was interpreted as arising from dairy fats (n=4), 1% as pig adipose fats (n=8) and the rest as ruminant carcass fats and mixtures between those products (Casanova et al., 2020). Compound-specific radiocarbon analyses (CSRA) of dairy lipids were revealed that dairy products were processed as early as 6087±27 BP (i.e. 5198-5158 cal BC, 3% probability or 5066-4939 cal BC, 93% probability) of the occurrence of milk products in the region (Casanova et al., 2020). Fracture and fragmentation of analysis of cattle bone suggests also marrow continued to be exploited throughout the Rubané (Johnston et al. 2018).

Černý Vůl (Czech Republic, LBK, 5200-5000 cal BC) is located in the basin of the Únětický stream. Rescue excavations from 1975 to 1977 revealed the ground plans of nine LBK post-hole houses dated to the LBK IIc-IIIb (Řídký et al., 2008). Cattle dominate the LBK faunal assemblage (43%; NISP=389), but caprines are well attested (20%). The cattle kill-off pattern resembles the pattern established at Chotěbudice, suggesting exploitation for milk and meat (Kovačiková et al., 2012). Calves in the 0-6 m age class are not represented, while the 6-15 m age class includes 8.7% of dental remains (N = 23; Gillis et al., 2017).

Cheia (Romania, Hamangia, 5215-4855 cal BC; Balasse et al., 2014) is a modest village in the Central Dobrudja plateau, between the Danube and the Black Sea in south-eastern Romania. It was periodically occupied by Hamangia communities at the turn of the 5th millennium cal BC, corresponding to the start of the Chalcolithic period in Dobrudja. Zooarchaeological analysis of the bone assemblage (mammals remains NISP=3,920) demonstrated a high predominance of husbandry (89%) over hunting and exploitation of cattle (49%) and caprines (sheep and goats, 39%) (Bălășescu and Radu, 2003, 2004; Bălășescu, 2008). No domestic pigs were positively identified. The cattle mortality profile suggests a mixed production of meat and milk. The 0-6 m and 5-13 age classes (following Jones & Sadler, 2012) include respectively 7 % and 12.8 % of cattle dental remains (N = 360). The caprines mortality profile shows an over-representation of individuals from the 6-12 months age class, suggesting an emphasis on tender meat production (Bălăşescu, 2008). Wild animal remains came from a wide spectrum consisting of a high percentage of red deer, a small size wild horse (probably hemione), aurochs and wild boar. The crop husbandry practices of these communities remain to be defined.

Hârşova-tell and **Borduşani-*Popină*** (Romania, Gumelniţa A2, 4340-4035 cal BC and 4490-4265 cal BC, respectively) are neighbouring sites located on the Danube River Plain, in south-eastern Romania. At both sites, Chalcolithic occupations from the Gumelniţa A2 culture were dated to the second half of the 5th millennium cal BC (Bréhard and Bălăşescu 2012; Gillis *et al.*, 2013; Bréhard et al., 2014). Evidence of agriculture has been recovered from both sites. The spectrum of cultivated plants is typical of the Chalcolithic period in south-eastern Europe, including varieties of wheat, naked barley and pulses. A substantial part of the subsistence economy derived from aquatic resources, while hunting targeted a variety of wild animals (Bălăşescu *et al.*, 2005). The importance of animal husbandry is highlighted by the clear predominance of domestic animals in the mammal assemblages. At Borduşani (large terrestrial mammals NISP=9,361), pig remains dominate the domestic animal component (41%), followed by cattle (21%) and caprines (17%, mostly sheep). At Hârşova (large terrestrial mammals NISP=5,640), caprines represent the highest number of domestic animals remains (26%, mostly sheep), with pigs a close second, then cattle (11%). At both sites, sheep were most likely exploited for meat - with a focus on early culling, while exploitation of milk is suggested from the cattle mortality profiles (Bréhard and Bălăşescu, 2012). Cattle mortality reaches 7.2 % in the 0-6 m age class and 19.2 % in the 6-15 m age class (N = 158; Bréhard and Bălăşescu, 2012). Milk was shared between herders and calves (Gillis et al., 2013). Stable isotope analysis of the bone remains revealed small-scale herding for cattle and caprines, reflected in an unexpectedly high contribution to their diet of ruderal C_4 plants, which was more abundant near the settlements and cultivated fields (Balasse *et al.*, 2017).

Auriac (France, southern Chasséen, 3986-3799 cal BC) is a large open-air settlement with an enclosure ditch, controlling a passage on the Aude river. The enclosed area is about four hectares. The site has yielded one of the most important series of artefacts for the classic Chasséen of the Languedoc region (Vaquer & Gandelin 2018). Most of the faunal remains come from the ditch and the dwelling area located inside the enclosure. The faunal assemblages (NISP=1,014 for the dwelling area) testify of the major role played by husbandry in subsistence. Domestic ruminants strongly dominated (90%), cattle and caprines (mostly sheep) being equally represented. The mortality profiles suggest mixed exploitations, for both milk and meat (Bréhard, 2011). Calves in the 0-6 m and 6-12 m age classes represent 7.9 % and 13.4 % of remains (N = 228; Bréhard, 2011)

Bercy (France, northern Chasséen, c. 3900 cal BC) is a prehistoric village adjacent to the Seine river (Paris, France) whose main period of occupation relates to the Chasséen septentrional culture (Marquis, 1991; Lanchon *et al.*, 2000). The animal subsistence economy (NISP=10410) relied heavily on cattle husbandry complemented by other species, including sheep. The hunted wild fauna, dominated by red deer, also included roe deer, aurochs, wild boar, brown bear and small (furry) game (Tresset, 1996a). The mortality profiles of the domestic animals indicate that the pigs and small stock were culled when they had reached a sufficient weight, suggesting a strategy essentially oriented

towards meat production. The cattle mortality profile suggests they were raised for meat, as evidenced by a slaughtering peak in the 2-4 year age class, and milk, as shown by a high proportion of calves in the 6-12 m age class (post-lactation slaughter) and the presence of individuals over 8 years (Tresset, 1996b; Balasse et al. 1997, 2000). Calf mortality in the 0-6 m and 6-15 m age classes reach respectively 10.4 % and 10.2 % (N = 231; Tresset, 1996b). A stable isotope study revealed that some cattle and sheep were fed on a ^{13}C-depleted resource in winter, probably leafy fodder collected/grazed in the dense oak tree forest covering the alluvial terrace (Balasse *et al.*, 2012).

References

Andreescu R., Mirea P. 2008. Teleorman Valley. The Beginning of Neolithic in Southern Romania. *Acta Terrae Septemcastrensis* **7**, 57–75.

Ashmore P. 2000. Dating the Neolithic in Orkney. In *Neolithic Orkney in its European context* (ed. A. Ritchie), 299-308, McDonald Institute for Archaeological Research, Cambridge, UK.

Ashmore P. 2009. Radiocarbon dates. In *On the fringe of Neolithic Europe. Excavation of a chambered cairn on the Holm of Papa Westray, Orkney* (ed. A. Ritchie), 59-66, Society of Antiquaries of Scotland, Edinburgh, UK.

Bălăşescu A. 2008. Considerații cu privire la exploatarea mamiferelor în așezarea. Hamangia III de la Cheia. *Pontica* **41**, 49-55.

Bălăşescu A. 2014. *Arheozoologia neo-eneoliticului de pe Valea Teleormanului, Muzeul Național de Istorie a României*. Colecția Muzeului Național, Seria Cercetări Puridisciplinare 14. Editura Mega, Cluj Napoca, Romania.

Bălăşescu A. and Radu, V. 2003. Studiul arheozoologic preliminar al materialului faunistic de la Cheia (jud. Constanța). Campania 2001. *Pontica* **33/34**, 25–30.

Bălăşescu A., Radu V. 2004. *Oameni și animale. Strategii și resurse la comunitățile preistorice Hamangia și Boian*. Seria Cercetări Pluridisciplinare 9. București: Biblioteca Muzeului Național, Editura Cetatea de Scaun.

Bălăşescu A., Radu V., Moise D. 2005. *Omul și mediul animal între mileniile VII-IV î.e.n. la Dunãrea de Jos. Muzeul National de Istorie a României*. Seria Cercetãri Pluridisciplinare 11. Editura Cetatea de Scaun, Târgoviște, Romania.

Balasse M., Bălăşescu A., Janzen A., Ughetto-Monfrin J., Mirea P., Andreescu R. 2013. Early herding at Măgura-Boldul lui Moş Ivănuş (early sixth millennium BC, Romania): environments and seasonality from stable isotope analysis. *Eur J. Archaeol.* **16(2)**, 221-246.

Balasse M., Bălăşescu A., Tornero C., Frémondeau D., Hovsepyan R., Gillis R., Popovici D. 2017. Investigating the scale of herding in Chalcolithic pastoral communities settled by the Danube river in the 5th millennium BC: a case study at Borduşani-Popină and Hârşova-tell (Romania). *Quat. Int.* **436B**, 29-40.

Balasse M., Bocherens H., Tresset A., Mariotti A., Vigne J.-D. 1997. Emergence de la production laitière au Néolithique? Contribution de l'analyse isotopique d'ossements de bovins archéologiques, *CRAS Paris, Sciences de la Terre et des planètes* **325**, 1005-1010.

Balasse M., Boury L., Ughetto-Monfrin J., Tresset A. 2012. Stable isotope insights ($\delta^{18}O$, $\delta^{13}C$) into cattle and sheep husbandry at Bercy (Paris, France, IVth millennium BC): birth seasonality and winter leaf foddering. *Env. Archaeol.* **17(1)**, 29-44.

Balasse M., Tornero C., Bréhard S., Ughetto-Monfrin J., Voinea V., Bălăşescu A. 2014. Cattle and sheep herding at Cheia, Romania, at the turn of the fifth millennium cal BC: a view from stable isotope analysis. In *Early farmers: the view from archaeology and science* (eds. A. Whittle, P. Bickle) 115-142, Proceedings of the British Academy.

Balasse M., Tresset A. 2009. A key to the adaptation of Neolithic husbandry in the Orkneys: contribution of seaweed to the sheep diet at the Holm of Papa Westray, revealed through stable isotope analysis ($\delta^{13}C$ and $\delta^{18}O$) of teeth. In *On the fringe of Neolithic Europe. Excavation of a chambered cairn on the Holm of Papa Westray, Orkney* (ed. A. Ritchie), 74-82, Society of Antiquaries of Scotland, Edinburgh, UK.

Balasse M., Tresset A., Ambrose S.H. 2006. First evidence for seaweed winter foddering in the Neolithic of Scotland. *J. Zool.* **270**, 170-176.

Balasse M., Tresset A., Bocherens H., Mariotti A., Vigne J.-D. 2000. Un abattage " post-lactation " sur des bovins domestiques néolithiques. Etude isotopique des restes osseux du site de Bercy (Paris, France). *Anthropozoologica* **31**, 39-48.

Bánffy, E., Höhler-Brockmann, H. 2020. Burnt daub talking: the formation of the LBK longhouse (a work hypothesis). *Quat. Int.*, June 2020 DOI: 10.1016/j.quaint.2020.06.007

Banffy E., Marton T., Osztas A. 2010. Early Neolithic settlement and burials at Alsonyek-Bataszek. In *Neolithization of the Carpathian Basin: Northernmost distribution of the Starcevo/Körös culture* (ed. J.K. Kozlowski, P. Raczky), 37-51, Polska Akademia Umiejętności, Kraków, Poland.

Bánffy E., Oross K. 2009. Entwicklung und Dynamik der Linearbandkeramik in Transdanubien. In *Krisen – Kulturwandel – Kontinuitäten: zum Ende der Bandkeramik in Mitteleuropa* (ed. A. Zeeb-Lanz), 219–40, Rahden: Marie Leidorf.

Bánffy E., Oross K. 2010. The earliest and earlier phase of the LBK in Transdanubia. In *Die Neolithisierung Mitteleuropas (The spread of the Neolithic to central Europe)* (eds. D. Gronenborn ,J. Petrasch), 255–72, Mainz: Römisch-Germanisches Zentralmuseum.

Bánffy E., Osztás A., Oross K., Zalai-Gaál I., Marton T., Nyerges É.Á., Köhler K., Bayliss A, Hamilton D., Whittle A. 2016. The Alsónyék story: towards the history of a persistent place. *Bericht der Römisch-Germanischen Kommission* **94**, 283-318.

Bem C., 2001. Noi propuneri pentru o schiță cronologică a eneoliticului românesc. *Pontica* **33-34**, 25-121.

Berthon R., Kovačiková L. Tresset A., Balasse M. 2018. Integration of Linearbandkeramik cattle husbandry in the forested landscape of the mid-Holocene climate optimum: Seasonal-scale investigations in Bohemia. *J. Anthropol. Archaeol.* **51**,16-27.

Biller, A. Zs. forthcoming: Animal keeping and hunting habits of the Early Neolithic settlement of Alsónyék in the mirror of the animal bones. In *The Neolithic of the Sárköz and adjacent regions in Hungary: bioarchaeological studies* (ed. E. Bánffy), Confinia et horizontes 2, Frankfurt am Main.

Bökönyi S. 1988. The Neolithic Fauna of Divostin. In *Divostin and the Neolithic of Central Serbia* (eds. A. McPherron, D. Srejović), 419-445, Department of Anthropology, University of Pittsburgh, Pittsburgh, USA.

Bréhard S. 2011. Le complexe chasséen vu par l'archéozoologie : révision de la dichotomie Nord-Sud et confirmation de la partition fonctionnelle au sein des sites méridionaux. *Bull. Soc. Préhist. Fr* **108**, 1 : 73-92.

Bréhard S., Bălăşescu A. 2012. What's behind the tell phenomenon? An archaeozoological approach of Eneolithic sites in Romania. *J. Archaeol. Sci.* **39**, 3167-3183.

Bréhard S., Radu V., Martin A., Hanot P., Popovici D., Bălăşescu A. 2014. Food supply strategies in the Romanian Eneolithic: sheep/goat husbandry and fishing activities from Hârşova tell and Borduşani-Popină (5th millennium BC). *Eur. J. Archaeol.* **17(3)**, 407-433.

Casanova E., Arbogast R.-M., Denaire A., Jeunesse C., Lefranc P., Evershed R. P. 2020. Spatial and temporal disparities in human subsistence in the Neolithic Rhineland gateway. *J. Archaeol. Sci.* **122**, 105215.

Clason A. 1980. Padina and Starčevo: game, fish and cattle. *Palaeohistoria* **22**, 142-173.

Clark D.V. 1976. *The Neolithic village at Skara Brae, Orkney: 1972-1973 excavations*. HMSO Edinburgh, UK.

Denaire A., Lefranc P., Wahl J., Bronk Ramsey C., Dunbar E., Goslar T., Bayliss A., Beavan N., Bickle P. Whittle A. 2017. The Cultural Project: formal chronological modelling of the Early and Middle Neolithic sequence in Lower Alsace. *J. Meth. Theor.* **24**, 1072–1149.

Domboróczki L., 2018. Preliminary data on the house structures of the LBK settlement at Apc-Berekalja (NE-Hungary). In *Multa per gentes et mutla per secula* (eds. Valde-Nowak P., Sobczyk K., Nowak M., Źrałka J.), 361-368, Kraków: Institute of Archaeology, Jagiellonian University.

Domboróczki L., Budek A., Daróczi-Szabó L., Kaczanowska M., Kalicki T., Klusakiewicz E., Kozłowski J.K., Kreuz A., Pomázi P., Wasilewski M., Zoffmann Z.K., 2016. Excavation along the easternmost frontier of the LBK in Ne-Hungary at APC-Berekalja I (2008-2009). *Archaeologiai Értesítő* **141**, 1–27.

Dreslerová, G. 2006. Vyhodnocení zvířecích kostí z neolitického sídliště Těšetice-Kyjovice (okr. Znojmo, Česká republika) (Die Auswertung der Tierknochen aus einer neolithischen Siedlung in Těšetice-Kyjovice (Bez. Znojmo, Tschechische Republik). *Archeologické rozhledy* **58(1)**, 3–32.

Ehrich R. 1977. Starčevo Revisited. In *Ancient Europe and the Mediterranean: Studies Presented in Honour of Hugh Hencken* (ed. V Markotic), 59-67, Aris & Phillips, Warminster.

Ethier J., Bánffy E., Vuković J., Leshtakov K., Bacvarov K., Roffet-Salque M., Evershed R., Ivanova M. 2017. Earliest expansion of animal husbandry beyond the Mediterranean zone in the sixth millennium BC. *Sci. Rep.* **7**, 7146. https://doi.org/10.1038/s41598-017-07427-x

Fewkes V., Goldman H., Ehrich R. 1933. Excavations at Starčevo, Yugoslavia, seasons 1931 and 1932. A preliminary report. *Bulletin / Am School Prehist Res* **9**, 32-54.

Gillis R., Bréhard S., Bălăşescu A., Ughetto-Monfrin J., Popivici D., Vigne J.-D., Balasse M. 2013. Sophisticated cattle dairy husbandry at Borduşani-Popină (Romania, fifth millennium BC): the evidence from complementary analysis of mortality profiles and stable isotopes. *World Archaeol* **45**, 447-472.

Gillis R.E., Gaastra J.S., Vander Linden M., Vigne J.D., 2019. A Species-Specific Investigation Into Sheep and Goat Husbandry During the Early European Neolithic, *Environ. Archaeol.* 10.1080/14614103.2019.1615214

Gillis R.E., Kendall I., Evershed R.P., Balasse M. in press. Conduite de l'élevage dans un environnement forestier à Bischoffsheim : une approche biogéochimique. In *Bischoffsheim : un village du Néolithique ancien en basse Alsace (France)* (eds. Lefranc Ph., Arbogast R.-M.), Archéologiques, Musée national d'Histoire et d'Art du Grand Duché du Luxembourg, Luxembourg.

Gillis R.E., Kovačiková L., Bréhard S., Guthmann E., Vostrovská I., Nohálová H., Arbogast R.-M., Domboroczki L., Pechtl J., Andres A., Marciniak A., Tresset A., Vigne J.-D. 2017. The evolution of dual meat and milk cattle husbandry in Linearbandkeramik societies. *Proc. R. Soc. B* **284**, 20170905.

Grbić M. 1930. Bemalte Keramik aus Starčevo im Banater Donaugelände-Jugoslavien. In *Księga pamiątkowa ku uczczeniu siedemdziesiaątej rocznicy urodzin prof. dr. Włodzimierza Demetrykiewicza* (ed. J Kostrzewski), 111-114, Nakładem Polskiego Towarzystwa Prehistorycznego, Poznan, Poland.

Greenfield H. 2000. Integrating surface and subsurface reconnaissance data in the study of stratigraphically complex sites: Blagotin, Serbia. *Geoarchaeol.* **15(2)**, 167-201.

Greenfield H., Jongsma T. 2006. The spatial organization of Early Neolithic settlements in temperate southeastern Europe: a view from Blagotin, Serbia. In *Space and Spatial Analysis in Archaeology* (eds. E. Robertson, J. Siebert, D. Fernandez, M. Zender), 69-79, University of Calgary Press, Calgary, Canada.

Greenfield H., Jongsma Greenfield T., Jazik S. 2014. Subsistence and settlement in the Early Neolithic of temperate SE Europe: a view from Blagotin, Serbia. *Archaeologia Bulgarica* **XVIII(1)**, 1-33.

Johnson E. V., Timpson A., Thomas M. G., Outram A. K. 2018. Reduced intensity of bone fat exploitation correlates with increased potential access to dairy fats in Early Neolithic Europe. *J. Archaeol. Sci.* **94**, 60–69.

Johnson, E.V., Gillis, R., Pyzel, J., Marciniak, A., Outram, A. K. 2021. The cattle of Ludwinowo 7: death, dinner and deposition in the Linearbandkeramik culture. In *Human and Cattle* (eds. E. Wright, C. Ginja), Lockwood Press, Atlanta. In press

Jones G.G., Sadler P. 2012. Age at death in cattle: methods, older cattle and known-age reference material. *Environ. Archaeol.* **17 (1)**, 11-28.

Kendall I., Gillis R., Balasse M., Evershed R. 2019. Stable isotope perspectives of cattle husbandry practices. In *Ludwinowo, site 7 Neolithic settlement in Kuyavia* (ed .J Pyzel), 277-288, Pekowice-Gdansk: University of Gdansk Publishing House.

Kovačiková L., Bréhard S., Šumberová R., Balasse M., Tresset A., 2012. The new insights into the subsistence and early farming from Neolithic settlements in Central Europe: the archaeozoological evidence from the Czech Republic. *Archaeofauna* **21**, 71–97.

Lanchon Y., Marquis P., Roblin-Jouve A. 2000. *Le Premier Village de Paris, il y a 6000 ans*. Les Découvertes Archéologiques de Bercy. Paris-Musées, Paris, France.

Leković V. 1988. Magareći mlin – Apatin. In *The Neolithic of Serbia: Archaeological Research 1948-1988* (ed. D Srejović), 24-38, Centre for Archaeological Research, Faculty of Philosophy, University of Belgrade, Belgrade, Serbia.

Marciniak A. 2005. *Placing Animals in the Neolithic: Social Zooarchaeology of Prehistoric Farming Communities*. London: UCL Press.

Marquis P. 1991. Le site néolithique de Bercy (Paris XIIe). *Bull. Soc. préhist. fr.* **88**, 239.

McPherron A., Aitken M., Bucha V. 1988. Absolute dating of Divostin, Grivac-Barice and Banja. In *Divostin and the Neolithic of Central Serbia* (eds. A McPherron, D Srejović), 379-387, Ethnology Monograps No 10. Department of Anthropology, University of Pittsburgh, Pittsburgh, USA.

McPherron A., Srejović D. (eds.) 1988. *Divostin and the Neolithic of Central Serbia*. Ethnology Monograps No 10. Department of Anthropology, University of Pittsburgh, Pittsburgh, USA.

Nowak M., Moskal-Del Hoyo M., Mueller-Bieniek A., Litynska-Zajac M., Kotynia K., 2017. Benefits and weaknesses of radiocarbon dating of plant material as reflected by neolithic archaeological sites from Poland, Slovakia and Hungary. *Geochronometria* **44**, 188-201.

Nyerges É.Á., Biller A.Z. 2015. Neolithic animal husbandry in the Tolnai-Sárköz Region on the basis of the archaeozoological finds from the Alsónyék–Bátaszék archaeological site. *Hungarian Archaeology E-Journal* 2015 Winter, 1-7. http://files.archaeolingua.hu/2015T/eng_Nyerges_15T.pdf

Oross K., Cramp L.J.E., Gortva G., Jakucs J., Lyublyanovics K., Marton T., Serlegi G., Vágvölgyi B., Whittle A., 2020. 'It's still the same old story': The current southern Transdanubian approach to the Neolithisation process of central Europe. *Quat. Int.* 560-561:154–178. https://doi.org/10.1016/j.quaint.2020.05.049

Osypińska M., Abłamowicz R. 2019. Animal exploitation and meat consumption model. Archaeozoological analysis of osteological materials from the LBK settlement. In *Ludwinowo 7 - Neolithic settlement in Kuyavia* (ed. Pyzel J.), 239-68, Gdańsk, Profil-Archeo Publishing House and Archaeological Studio University of Gdańsk Publishing House.

Osztás A., Zalai-Gaál I., Bánffy E. 2012. Alsónyék-Bátaszék: a new chapter in the research of Lengyel culture. *Documenta Praehistorica* **39**, 377–396.

Pinhasi R., Fort J., Ammerman A. 2005. Tracing the origin and spread of agriculture in Europe. *PLoS Biology* **3 (12)**, 2220–2228.

Pleinerová I Klementová J, Hložek J, Daněček D 2005. *Záchranný archeologický výzkum v lokalitě Sadová I.+II., Hostivice, k.ú. Hostivice. Zprávy České archeologické společnosti.*

Porčić M., Blagojević T., Pendić J., Stefanović S. 2020. The timing and tempo of the Neolithic expansion across the Central Balkans in the light of the new radiocarbon evidence. *J. Archaeol. Sci. Reports* **33**, 102528. https://doi.org/10.1016/j.jasrep.2020.102528

Porčić M., Blagojević T., Pendić J., Stefanović S. 2021. The Neolithic Demographic Transition in the Central Balkans: population dynamics reconstruction based on new radiocarbon evidence. *Philos. Trans. R. Soc. B: Biological Sciences* **376**, 20190712. http://dx.doi.org/10.1098/rstb.2019.0712

Pyzel J. 2019. Radiocarbon dating of the Neolithic occupation, in *Ludwinowo 7 - Neolithic settlement in Kuyavia* (ed. Pyzel), 331-5, Gdańsk, Profil-Archeo Publishing House and Archaeological Studio University of Gdańsk Publishing House.

Pyzel J., Pilarski B., Cyganiewicz P. 2019. LBK non-portable features. In *Ludwinowo 7 - Neolithic settlement in Kuyavia* (ed. Pyzel J.), 19-42, Gdańsk, Profil-Archeo Publishing House and Archaeological Studio University of Gdańsk Publishing House.

Rada I., 1981. The investigation of the Linear Pottery settlement at Chotěbudice in 1974. *Archeologické rozhledy* **3**3, 3–18.

Rassmann K., Mischka C., Furholt M., Ohlrau R., Radloff K., Winkelmann K., Serlegi G., Marton T., Osztás A., Oross K. and Bánffy E. 2015. Large scale geomagnetic prospection on neolithic sites in *Hungary, Hungarian Archaeology E-Journal* 2015 Spring, 1-8 http://files.archaeolingua.hu/2015TA/Rassmann_E15TA.pdf

Řídký J., Stolz D., Zápotocká M., 2008. Neolithic Settlement in Černý Vůl (Prague-west). Forms of features, pottery analysis and chipped stone industry from excavations in 1975–77 and 1914. *Praehistorica* **28**, 177–236.

Ritchie A. 1984. Excavation of a Neolithic farmstead at Knap of Howar, Papa Westray, Orkney. *Proceedings of the Society of Antiquaries of Scotland* **113**, 40-121.

Ritchie A. 2009. *On the fringe of Neolithic Europe. Excavations of a chambered cairn on the Holm of Papa Westray, Orkney*. Society of Antiquaries of Scotland, Edinburgh, UK.

Roffet-Salque M., Regert M., Evershed R. *et al.* Widespread exploitation of the honeybee by early Neolithic farmers. *Nature* **527,** 226–230 (2015). https://doi.org/10.1038/nature15757

Roffet-Salque, M., Banecki, B., Krüger, M., Pyzel, J., Smyth, J., Evershed Richard, P. 2019. Chronological and spatial trends in pottery use revealed through lipid residue analyses of LBK pottery vessels. In *Ludwinowo 7 - Neolithic settlement in*

Kuyavi (ed. J. Pyzel), 301-316, Gdańsk, Poland, Profil-Archeo Publishing House and Archaeological Studio University of Gdańsk Publishing House.

Salque M., Bogucki P. I., Pyzel J., Sobkowiak-Tabaka I., Grygiel R., Szmyt M., Evershed R. P. 2013. Earliest evidence for cheese making in the sixth millennium BC in northern Europe. *Nature* **493**, 522-5.

Stanković S., Greenfield H. 1992. Arheološka istraživanja višeslojnog praistorijskog lokaliteta Blagotin u selu Poljna (iskopavanja 1991. godine). *Glasnik Srpskog arheološkog društva* **8**, 46-49.

Stanković S., Leković V. 1993. Neolithic settlement at Blagotin. *Glasnik Srpskog arheološkog društva* **9**, 177-179.

Stefanović S., Petrović B., Porčić M., Penezić K., Pendić J., Dimitrijević V., Živaljević I., Vuković S., Jovanović J., Kojić S., Starović A., Blagojević T. 2019. Bone spoons for prehistoric babies: Detection of human teeth marks on the Neolithic artefacts from the site Grad-Starčevo (Serbia). *PLoS ONE* **14(12)**, e0225713.

Stojanovski D., Živaljević I., Dimitrijević V., Dunne J., Evershed R., Balasse M., Dowle A., Hendy J., McGrath K., Fischer R., Speller C., Jovanović J., Casanova E., Knowles T., Balj L., Naumov G., Putica A., Starović A., Stefanović S. 2020. Living off the land: Terrestrial-based diet and dairying in the farming communities of the Neolithic Balkans. *PLoS ONE* **15(8)**, e0237608. https://doi.org/10.1371/journal.pone.0237608

Šumberová R., 1991. Neolithic settlement site at Chotěbudice, Distr. of Louny (excavations of 1988 and 1990). In *Archaeology in Bohemia 1986-1990* (eds. Charvát P., Neústupný E., Vařeka P.), 194-195, Institute of Archaeology of the Czechoslovak Academy of Sciences, Prague.

Tasić, N. 1993. Nekoliko novih radiokarbonskih datuma sa lokaliteta Deronje i Magareći mlin. *Glasnik Srpskog arheološkog društva* **9**, 99-102.

Tresset A. 1996a *Quelques aspects de l'approvisionnement carné en contexte Chasséen septentrionnal à Bercy*. Unpublished report. SRA Ile de France.

Tresset A. 1996b. *Le Rôle des Relations Homme/Animal dans l'Evolution Economique et Culturelle des Sociétés des V-IVe Millénaires en Bassin Parisien*. Thèse de doctorat, Université Paris 1, Paris, France.

Tresset A. 2003. French connections II: of cows and men. In *Neolithic settlement in Ireland and western Britain* (ed. I. Armit, E. Murphy, E.L. Nelis, D.D.A. Simpson), 18-30, Oxbow Books, Oxford.

Vaquer J., Gandelin M., 2018, Auriac à Carcassonne (Aude). Une enceinte du Chasséen méridional. In *Les sites ceinturés de la Préhistoire récente. Nouvelles données, nouvelles approches, nouvelles hypothèses. Actes de la table ronde de Carcassonne des 12 et 13 octobre 2012 et travaux du projet collectif de recherche "Milieu et Peuplement en Languedoc occidental du Néolithique au Bronze ancien"* (eds. Gandelin M., Ard V., Vaquer J., Jallot L.), 33-54, Archives d'Ecologie Préhistorique, Toulouse.

Vostrovská I., 2018. *Těšetice-Kyjovice. Komunitní areál prvních zemědělců*. PhD Thesis. Masaryk University, Brno, https://is.muni.cz/th/f7qiy/Vostrovska_2018_DP. pdf accessed on December 17, 2020.

Whittle A., Bartosiewicz L., Borić D., Pettitt P., Richards M. 2002. In the beginning: new radiocarbon dates for the Early Neolithic in northern Serbia and south-east Hungary. *Antaeus* **25(2002)**, 63-117.

Whittle A.W.R., Bentley R.A., Bickle P., Dočkalová M., Fibiger L., Hamilton J., Hedges R.E.M., Mateiciucová I., Pavúk J., 2013. 4. Moravia and western Slovakia. In *LBK lifeways: a search for differences* (eds. Whittle, A.W.R., Bickle, P.), 101-158, Oxbow, Oxford.

Živković M., Vukadinović M., Antonović D. 2011. Geofizička i arheološka istraživanja u Starčevu: novi pristup zaštiti neolitskog naselja. *Rad Muzeja Vojvodine* **5**3, 7-22.

Supplementary Table S3: Results from the sequential analysis of enamel δ^{18}O values: Balatonszárszó (Hungary)

BAL Bos1 M3		BAL Bos2 M3		BAL Bos3 M3		BAL Bos4 M3		BAL Bos5 M3	
distance (mm)	δ^{18}O (‰)	distance (mm)	δ^{18}O (‰)	distance (mm)	δ^{18}O (‰)	distance (mm)	δ^{18}O (‰)	distance (mm)	δ^{18}O (‰)
50.8	-6.73	48.2	-6.07	50.3	-6.95	46.1	-5.97	47.9	-8.05
47.3	-7.08	45.1	-6.31	46.0	-7.45	42.6	-6.43	45.2	-7.97
44.1	-6.08	42.1	-7.81	43.5	-8.20	38.7	-6.68	43.3	-7.75
41.5	-5.57	38.5	-8.15	40.0	-8.61	35.8	-6.69	41.1	-7.06
37.6	-4.43	35.3	-8.40	37.1	-8.62	32.5	-6.24	37.6	-5.97
34.5	-3.79	32.3	-8.28	34.1	-8.22	29.3	-5.61	34.2	-5.05
30.7	-3.08	29.3	-7.65	30.8	-7.70	26.6	-5.02	30.4	-4.72
27.8	-3.50	26.3	-6.84	27.5	-7.16	24.0	-4.02	27.2	-4.20
25.1	-3.96	22.4	-5.46	24.7	-6.64	20.9	-3.05	24.6	-4.62
21.9	-5.21	19.1	-3.84	22.2	-6.60	17.2	-2.35	21.4	-5.21
18.1	-5.93	15.6	-3.68	18.7	-6.05	13.5	-2.20	18.5	-6.12
15.7	-6.69	12.5	-3.17	16.6	-5.55	11.3	-2.11	14.9	-6.47
12.1	-7.64	9.5	-3.91	14.5	-5.62	9.1	-2.39	11.8	-6.74
9.6	-7.99	6.7	-4.43	12.6	-5.65	6.2	-2.90	8.8	-6.42
6.7	-7.18	3.9	-5.64	10.5	-5.89	3.1	-3.56	4.7	-5.99
3.5	-5.16			8.3	-6.58				
				6.5	-6.86				
				4.5	-7.68				

Supplementary Table S4: Results from the sequential analysis of enamel $\delta^{18}O$ values at Apc (Hungary)

APC Bos1 M3		APC Bos2 M3		APC Bos3 M3		APC Bos4 M3		APC Bos5 M3		APC Bos6 M3		APC Bos7 M3*		APC Bos8 M3	
(mm)	$\delta^{18}O$ (‰)	(mm)	$\delta^{18}O$ (‰)	(mm)	$\delta^{18}O$ (‰)	(mm)	$\delta^{18}O$ (‰)	(mm)	$\delta^{18}O$ (‰)	(mm)	$\delta^{18}O$ (‰)	(mm)	$\delta^{18}O$ (‰)	(mm)	$\delta^{18}O$ (‰)
48.9	-3.57	43.5	-8.91	54.7	-7.74	43.4	-6.41	38.9	-8.69	32.7	-9.42	36.7	-8.08	31.5	-7.89
46.3	-4.40	41.0	-8.71	52.5	-7.44	42.7	-6.15	37.6	-8.34	31.2	-8.96	35.1	-7.60	28.9	-7.58
44.4	-5.57	39.1	-8.38	49.5	-7.64	41.5	-5.26	35.4	-8.07	29.9	-8.86	32.4	-6.78	27.0	-7.97
42.5	-6.20	37.1	-8.23	47.3	-7.21	38.3	-4.76	33.7	-7.62	27.3	-8.52	30	-6.48	25.5	-8.08
40.9	-6.95	35.1	-7.72	45.5	-6.55	35.5	-4.39	31.7	-6.99	25.7	-7.72	27.6	-6.08	23.4	-7.60
38.7	-7.75	33.3	-7.67	43.1	-5.84	33.0	-4.76	28.9	-6.62	23.4	-7.24	25.5	-5.79	20.6	-7.10
36.7	-8.56	30.2	-7.00	40.2	-4.66	31.3	-4.92	26.1	-6.37	20.2	-6.69	22.6	-5.73	18.5	-6.38
33.8	-8.43	29.9	-6.86	36.5	-4.21	29.0	-5.38	24.7	-5.50	18.1	-6.20	21.2	-5.57	15.9	-5.30
31.4	-8.52	28.5	-6.23	33.4	-3.95	25.7	-5.68	21.2	-5.55	16.4	-6.18	18.6	-5.73	9.0	-4.34
29.5	-8.64	26.0	-6.17	30.9	-4.53	23.8	-6.23	19.1	-5.37	13.3	-6.38	15.9	-6.00	7.0	-4.39
28.0	-7.52	24.0	-6.09	27.8	-4.78	21.5	-6.68	17.0	-5.34	10.8	-6.53	14.1	-6.31	4.5	-3.96
25.5	-6.54	20.4	-5.96	25.4	-5.46	18.9	-7.02	14.2	-5.78	8.8	-7.20	11.8	-6.29	3.4	-5.01
22.9	-5.91	17.7	-6.36	23.4	-5.68	16.2	-6.70	13.2	-6.35	6.8	-7.58	9	-6.74	2.4	-3.86
20.7	-5.00	15.5	-6.71	20.3	-6.39	13.5	-5.91	10.3	-6.77	4.9	-8.04	7	-6.52	1.1	-4.56
18.3	-4.41	12.8	-7.24	17.8	-6.53	11.7	-5.03	7.4	-7.57			4.1	-6.78		
15.2	-3.67	10.3	-7.99	15.7	-6.75			5.3	-8.33						
13.1	-3.06	8.3	-8.29	12.4	-7.35			4.1	-8.53						
10.0	-2.89	5.1	-8.40	9.0	-7.01										
7.2	-3.57			6.6	-5.96										
5.6	-3.72			4.2	-4.67										

* the $\delta^{18}O$ sequence could not be modeled

Supplementary Table S5: Results from the sequential analysis of enamel δ^{18}O values at Starčevo-Grad (LBSG), Magareći mlin (LBMM), Divostin (LBDI), Blagotin (BL) (Serbia)

**BL Bos1 M3**		**BL Bos2 M3**		**BL Bos4 M3**		**BL Bos5 M3**		**LBSG 074 M3**		**LBMM 025 M3**		**LBMM 023 M3**		**LBMM045 M3**		**LBDI001 M3**	
distance (mm)	δ^{18}O (‰)	distance (mm)	δ^{18}O (‰)	distance (mm)	δ^{18}O (‰)	distance (mm)	δ^{18}O (‰)	distance (mm)	δ^{18}O (‰)	distance (mm)	δ^{18}O (‰)	distance (mm)	δ^{18}O (‰)	distance (mm)	δ^{18}O (‰)	distance (mm)	δ^{18}O (‰)
44.8	-6.23	45.2	-6.68	45.7	-8.04	48.6	-8.18	46.0	-5.64	50.5	-6.41	39.9	-9.48	48.8	-6.24	49.5	-5.95
42.7	-6.50	43.1	-7.07	43.7	-8.34	46.5	-8.30	43.7	-7.53	48.9	-6.69	37.1	-8.78	47.3	-7.10	47.6	-6.47
40.7	-6.54	41.0	-7.26	41.8	-8.37	44.4	-8.50	41.6	-8.02	46.7	-7.27	34.8	-8.40	45.1	-7.18	43.8	-6.96
38.4	-6.74	39.1	-7.56	39.6	-8.45	42.4	-8.25	39.5	-8.55	45.0	-7.50	32.7	-7.56	43.2	-7.98	41.3	-7.57
36.3	-6.77	37.1	-7.57	37.8	-8.60	40.2	-8.36	37.1	-8.65	43.1	-7.54	31.3	-7.70	40.9	-7.98	38.5	-7.79
34.1	-6.59	35.0	-7.75	36.2	-8.70	38.3	-8.22	35.6	-8.89	41.4	-7.85	29.1	-6.61	38.7	-8.05	36.6	-8.57
31.8	-6.41	32.8	-7.60	34.1	-8.38	36.1	-8.03	33.5	-9.01	39.5	-7.79	26.9	-6.49	36.2	-7.11	34.2	-8.04
29.6	-6.05	30.8	-7.53	32.3	-8.19	34.0	-7.96	31.9	-8.88	37.8	-8.22	25.1	-6.00	33.3	-7.37	32.9	-8.26
27.5	-5.76	28.6	-7.29	30.3	-8.13	32.0	-7.74	29.3	-8.17	36.0	-8.36	22.4	-5.65	30.6	-6.21	30.4	-8.33
25.6	-5.74	26.4	-7.02	28.6	-7.79	29.8	-7.41	28.1	-7.48	34.2	-8.24	20.5	-5.49	27.9	-5.76	28.1	-7.98
23.3	-5.18	24.3	-6.63	26.8	-7.23	27.6	-7.51	26.3	-6.75	32.5	-7.98	18.6	-5.34	25.6	-4.75	26.1	-7.72
21.0	-5.28	22.2	-6.46	24.8	-7.03	25.9	-7.41	24.4	-6.14	30.4	-7.60	16.7	-5.98	23.6	-4.47	23.6	-7.81
19.0	-4.89	20.3	-5.96	22.6	-6.68	23.8	-7.46	22.3	-5.20	28.2	-6.98	14.8	-6.19	21.4	-3.86	21.3	-7.48
16.8	-5.12	18.3	-6.04	20.8	-6.51	21.6	-7.35	20.3	-4.95	26.4	-6.25	12.8	-6.99	19.4	-4.14	18.1	-7.21
14.7	-5.16	16.1	-5.88	18.8	-6.10	19.7	-7.31	18.8	-4.04	25.0	-5.69	11.2	-7.80	18.0	-4.32	16.6	-6.73
12.5	-5.66	14.3	-6.32	16.7	-6.13	17.8	-7.54	16.7	-3.27	23.3	-5.18	9.2	-8.02	16.4	-4.46	14.9	-7.02
10.5	-5.89	12.3	-6.39	14.7	-6.05	15.7	-7.69	14.9	-2.77	21.1	-4.78	7.1	-8.55	13.0	-5.10	12.4	-6.86
8.5	-6.39	10.1	-7.04	12.8	-6.51	13.6	-7.82	12.5	-3.00	19.3	-4.30	5.6	-9.14	11.8	-5.81	10.6	-7.42
6.5	-6.86	7.9	-7.40	11.0	-6.72	11.6	-7.89	10.3	-3.51	17.1	-3.73	3.6	-8.67	9.3	-6.49	8.8	-7.55
4.1	-7.59	5.9	-8.10	9.2	-7.33	9.9	-8.01	8.0	-4.00	15.8	-3.95	2.0	-8.18	7.1	-7.18	6.3	-7.97
		3.7	-8.57	7.2	-7.41	8.0	-8.23	6.1	-4.29	13.2	-4.02			5.3	-7.03	4.7	-8.26
				5.5	-7.79	6.0	-8.30	3.9	-4.60	11.5	-5.03			3.3	-6.99		
										9.3	-5.31						
										7.3	-6.43						
										5.2	-7.15						
										3.5	-7.51						

Supplementary Table S6: Results from the sequential analysis of enamel $\delta^{18}O$ values at Těšetice-Kyjovice (Czech Republic)

TSE Bos1 M3*		TSE Bos2 M3	
distance (mm)	$\delta^{18}O$ (‰)	distance (mm)	$\delta^{18}O$ (‰)
43.03	-9.36	44.3	-7.8
37.55	-8.47	42.8	-7.6
35.72	-6.42	40.4	-7.9
32.97	-5.17	39.4	-7.8
29.92	-4.71	36.5	-7.7
27.22	-5.07	34.8	-7.5
26.12	-5.91	32.6	-7.3
19.8	-7.06	30.5	-6.2
16.6	-7.72	28.0	-6.0
12.7	-8.33	26.6	-5.2
10.48	-8.09	23.7	-5.1
		22.2	-4.4
		19.5	-4.2
		16.7	-3.6
		15.2	-4.3
		13.1	-4.5
		11.0	-5.3
		9.9	-5.7
		7.7	-6.3
		5.0	-6.4

* the $\delta^{18}O$ sequence could not be modeled

Supplementary Table S7 Results from the sequential analysis of enamel δ^{18}O values at Borduşani (Romania)

PBORD BosM3sup1		PBORD BosM3sup2		PBORD BosM3sup3		PBORD BosM3sup4	
distance (mm)	δ^{18}O (‰)	distance (mm)	δ^{18}O (‰)	distance (mm)	δ^{18}O (‰)	distance (mm)	δ^{18}O (‰)
39.4	-7.24	41.3	-5.74	39.9	-8.37	48.3	-4.98
37.4	-7.40	39.1	-5.73	37.8	-8.33	46.0	-4.96
35.2	-7.19	37.0	-5.64	36.0	-7.97	43.8	-5.09
32.8	-7.28	34.7	-6.06	33.9	-8.00	41.6	-5.30
30.8	-6.53	32.5	-6.19	31.9	-7.56	39.6	-5.23
28.8	-6.27	30.5	-6.47	30.1	-7.46	37.4	-5.49
26.8	-5.54	28.4	-6.93	28.3	-6.67	35.4	-5.98
24.5	-5.14	26.3	-7.16	25.6	-6.50	33.0	-6.08
22.5	-4.93	24.1	-7.25	23.7	-6.19	30.8	-6.63
20.6	-5.07	21.8	-7.85	21.8	-6.55	28.5	-6.80
18.2	-5.12	19.3	-7.93	20.0	-6.86	26.6	-7.11
16.2	-5.62	17.1	-8.08	15.9	-7.64	24.3	-7.72
14.2	-5.81	14.9	-7.91	13.8	-8.32	22.2	-7.95
11.9	-6.38	12.5	-7.87	11.7	-8.48	20.0	-8.15
9.6	-6.95	10.3	-7.25	9.6	-8.68	17.7	-8.31
7.5	-7.39	8.0	-6.78	7.5	-8.82	15.4	-8.00
5.5	-7.27	5.7	-6.26	5.4	-8.73	13.2	-7.98
3.5	-7.01	3.5	-5.67	3.6	-8.03	10.8	-7.58
						8.2	-7.34
						5.9	-6.45
						3.8	-5.86
						1.5	-5.86

Supplementary Table S8: Results from the sequential analysis of enamel δ^{18}O values at Auriac (France)

AUR Bos1 M3		AUR Bos2 M3		AUR Bos3 M3		AUR Bos4 M3*		AUR Bos5 M3		AUR Bos6 M3		AUR Bos7 M3		AUR Bos8 M3		AUR Bos9 M3	
distance (mm)	δ^{18}O (‰)	distance (mm)	δ^{18}O (‰)	distance (mm)	δ^{18}O (‰)	distance (mm)	δ^{18}O (‰)	distance (mm)	δ^{18}O (‰)	distance (mm)	δ^{18}O (‰)	distance (mm)	δ^{18}O (‰)	distance (mm)	δ^{18}O (‰)	distance (mm)	δ^{18}O (‰)
38.2	-3.58	46.4	-3.64	46.2	-2.97	32.8	-2.31	46.4	-2.65	48.6	-3.74	38.8	-4.76	42.4	-4.14	42.3	-3.70
35.9	-3.82	44.2	-3.85	44.0	-3.06	30.6	-2.70	44.2	-3.19	46.8	-3.91	36.9	-4.83	40.3	-4.36	40.0	-3.91
33.7	-4.01	42.1	-4.04	42.1	-3.37	28.4	-3.10	41.9	-3.41	44.8	-4.27	34.8	-5.55	38.0	-4.41	37.9	-3.90
31.2	-4.05	40.1	-3.95	39.8	-3.28	26.2	-3.55	39.7	-3.45	42.5	-4.65	32.7	-5.21	35.9	-4.67	35.6	-3.90
29.0	-3.94	38.0	-3.87	38.1	-3.22	23.8	-4.01	37.5	-3.72	40.4	-4.69	30.5	-5.41	33.6	-4.67	33.0	-3.92
27.1	-3.84	35.8	-3.61	35.9	-3.64	22.0	-4.20	35.3	-3.99	38.2	-4.81	28.3	-5.61	31.7	-4.84	30.6	-3.61
25.1	-3.47	33.7	-3.73	33.7	-3.32	19.9	-4.48	33.3	-4.25	36.1	-4.64	26.1	-5.44	29.7	-4.56	28.5	-3.39
23.0	-3.33	31.8	-3.36	31.6	-3.46	17.5	-4.75	31.0	-4.17	33.7	-4.75	23.8	-5.38	27.5	-4.04	26.6	-3.46
21.1	-2.94	29.6	-3.20	29.9	-3.34	15.2	-4.85	28.4	-4.37	31.5	-4.80	21.6	-5.12	25.5	-3.86	24.2	-2.90
19.1	-2.62	27.4	-3.07	27.8	-3.06	13.1	-4.96	26.3	-4.30	29.3	-4.79	19.3	-4.50	23.1	-2.98	22.1	-2.78
16.9	-2.06	25.1	-2.59	26.0	-2.88	11.0	-4.61	24.3	-4.35	26.7	-4.61	17.2	-4.21	20.9	-2.52	20.0	-2.44
14.6	-1.87	23.0	-2.67	23.4	-2.65	9.1	-3.92	22.0	-4.18	24.3	-4.26	15.0	-3.89	18.6	-1.80	17.7	-2.46
12.2	-1.89	20.9	-2.61	21.2	-2.55	6.8	-3.41	19.9	-4.13	22.1	-4.43	12.9	-3.95	16.6	-1.71	15.5	-2.43
10.3	-2.06	18.7	-2.79	19.3	-2.38	4.6	-3.23	17.5	-3.78	19.8	-4.01	10.9	-3.46	14.4	-1.09	13.4	-2.38
8.4	-2.02	16.6	-3.00	17.2	-2.11	2.7	-2.83	15.4	-3.56	17.3	-3.90	8.7	-3.72	12.3	-1.59	11.4	-2.69
6.4	-2.29	14.5	-3.26	15.1	-2.36			13.3	-3.22	14.7	-3.65	6.6	-3.95	10.3	-1.26	9.2	-2.90
4.2	-2.29	12.7	-3.49	12.9	-2.74			10.9	-3.01	12.2	-3.80	4.5	-4.10	8.3	-1.64	7.4	-3.18
		10.4	-3.83	10.8	-2.92			8.6	-2.84	9.7	-3.92	2.6	-4.09	6.2	-2.27	5.4	-3.65
		8.3	-4.23	8.7	-3.27			6.4	-3.30	7.4	-3.95			4.1	-3.04	3.2	-3.81
		6.5	-4.23	6.4	-3.62			4.0	-3.27	5.3	-4.48						
		4.5	-4.40	4.6	-3.74					3.1	-4.62						

* the δ ^{8}O sequence could not be modeled

Supplementary Table S9: Results from the sequential analysis of enamel $\delta^{18}O$ values at Alsónyék (Hungary). All upper teeth.

ALS Bos1 M3		ALS Bos2 M3		ALS Bos3 M3		ALS Bos4 M3		ALS Bos5 M3		ALS Bos6 M3		ALS Bos7 M3		ALS Bos8 M3		ALS Bos9 M3		ALS Bos10 M3		ALS Bos11 M3	
mm	$\delta^{18}O$ (‰)	mm	$\delta^{18}O$ (‰)	mm	$\delta^{18}O$ (‰)	mm	$\delta^{18}O$ (‰)	mm	$\delta^{18}O$ (‰)	mm	$\delta^{18}O$ (‰)	mm	$\delta^{18}O$ (‰)	mm	$\delta^{18}O$ (‰)	mm	$\delta^{18}O$ (‰)	mm	$\delta^{18}O$ (‰)	mm	$\delta^{18}O$ (‰)
49.1	-4.26	45.6	-3.91	40.2	-8.13	50.4	-2.06	49.4	-6.96	52.0	-7.01	47.3	-5.28	48.2	-5.12	49.7	-5.99	48.8	-6.17	45.2	-3.84
47.1	-4.08	43.2	-3.68	38.5	-7.92	48.6	-1.69	47.6	-6.84	50.5	-7.15	44.7	-4.90	45.8	-4.53	47.3	-6.00	46.3	-5.80	43.1	-3.95
45.0	-3.89	41.1	-3.40	36.3	-7.71	46.5	-1.46	45.7	-6.18	48.6	-7.31	42.7	-4.69	43.4	-4.33	45.2	-6.02	44.2	-5.30	40.6	-4.32
42.7	-3.48	39.0	-3.44	34.0	-7.55	44.7	-1.68	44.0	-6.32	46.7	-7.42	40.6	-4.32	40.9	-4.25	43.1	-5.89	41.9	-4.49	38.4	-4.78
40.5	-3.19	36.8	-2.75	32.0	-6.92	42.9	-1.68	42.0	-5.76	44.9	-7.34	38.4	-4.21	38.6	-4.37	40.8	-5.95	39.5	-4.20	36.1	-4.93
38.3	-3.94	34.6	-2.90	29.9	-6.46	40.8	-1.87	40.6	-5.63	43.2	-7.26	36.4	-3.77	36.5	-4.81	38.8	-5.73	37.3	-3.99	33.7	-5.74
36.2	-4.02	32.2	-3.42	28.0	-5.60	39.0	-2.41	38.6	-5.46	41.2	-7.08	34.0	-3.87	34.3	-5.19	36.2	-5.72	35.0	-3.80	31.4	-6.11
34.1	-4.33	30.1	-3.79	25.9	-5.38	36.7	-3.48	36.6	-5.44	39.5	-6.58	31.6	-3.77	32.1	-5.57	34.1	-5.35	32.6	-3.20	29.1	-6.60
32.1	-4.64	27.7	-4.29	24.0	-4.48	35.2	-3.65	34.8	-5.54	37.6	-6.46	29.9	-3.77	30.0	-5.76	31.9	-4.98	30.2	-3.42	26.7	-7.22
29.9	-4.94	25.7	-5.00	22.0	-4.37	33.1	-4.34	33.1	-5.95	35.9	-5.95	28.0	-4.14	27.6	-6.18	29.2	-4.52	27.9	-3.47	24.6	-7.45
27.7	-5.86	23.6	-5.09	20.1	-4.06	31.3	-5.40	31.3	-6.01	34.0	-5.60	25.7	-4.63	25.6	-6.68	26.8	-4.27	25.9	-3.85	22.1	-7.31
25.7	-6.12	21.4	-5.65	18.1	-4.78	29.7	-6.04	29.4	-6.37	31.9	-5.59	23.8	-4.91	23.1	-6.63	24.5	-4.12	23.6	-4.63	19.9	-7.60
23.5	-6.84	19.5	-6.29	16.0	-5.25	27.8	-6.50	27.7	-6.66	29.9	-5.72	21.9	-5.51	20.9	-6.84	22.0	-3.80	21.1	-5.66	17.3	-7.16
21.3	-7.01	17.5	-6.48	14.0	-5.91	25.9	-7.39	26.1	-6.73	27.7	-5.73	19.8	-5.64	18.5	-6.45	19.7	-3.69	19.0	-5.96	14.8	-6.72
19.2	-6.57	15.5	-7.08	11.9	-5.46	24.1	-7.40	24.6	-6.78	25.7	-5.92	17.6	-6.10	15.8	-6.06	17.5	-4.05	16.6	-6.71	12.6	-6.25
16.9	-6.83	13.2	-7.29	9.8	-6.58	22.3	-8.16	22.8	-6.95	23.7	-6.17	15.4	-6.54	13.3	-5.74	14.9	-4.21	14.1	-7.05	10.3	-5.60
14.7	-5.90	11.2	-7.25	7.8	-7.18	20.4	-8.18	21.1	-7.45	21.6	-6.44	13.5	-7.19	11.0	-4.65	12.8	-4.53	12.2	-6.82	7.8	-5.47
12.5	-5.11	8.8	-7.16	5.8	-8.21	18.5	-8.68	19.3	-7.91	19.6	-6.64	11.4	-7.19	8.7	-3.95	10.2	-4.94	9.7	-7.03	5.6	-5.52
10.2	-4.50	6.4	-7.30	3.8	-7.99	16.5	-8.85	17.5	-7.91	17.5	-7.15	9.4	-7.82	6.1	-3.99	8.1	-5.58	7.6	-6.28	3.7	-5.82
8.0	-3.74	4.3	-6.19	2.0	-7.77	14.6	-9.10	15.5	-8.40	15.3	-7.39	7.5	-7.78	4.0	-3.99	5.8	-6.22	5.5	-5.64		
5.7	-3.51					12.6	-7.93	13.6	-8.68	13.3	-7.79	5.4	-7.32								
3.7	-2.86					10.7	-8.03	11.5	-8.60	11.2	-8.06	3.8	-6.21								
						8.5	-5.91	9.6	-8.09	9.2	-8.49										
						6.4	-4.67	7.5	-7.94	6.9	-8.40										
						4.3	-2.95	5.5	-7.04	4.5	-8.09										

Supplementary Figure S1: Results from the sequential analysis of enamel $\delta^{18}O$ values at Balatonszárszó (Hungary).

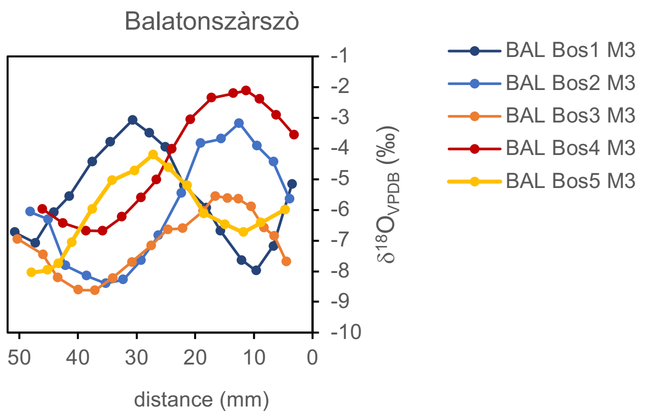

Supplementary Figure S2: Results from the sequential analysis of enamel $\delta^{18}O$ values at Apc (Hungary)

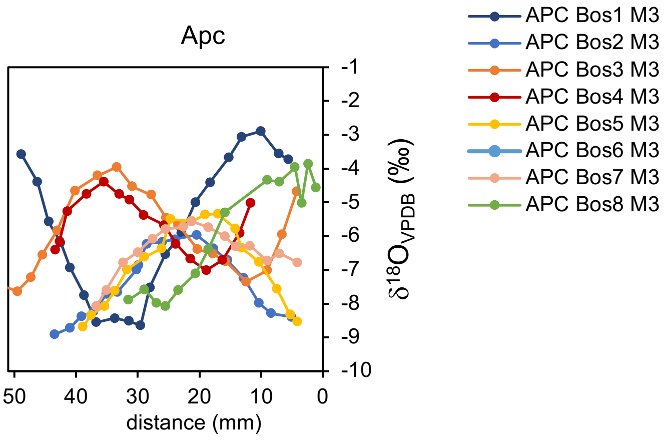

Supplementary Figure S3: Results from the sequential analysis of enamel $\delta^{18}O$ values at Starčevo-Grad (LBSG). Magareći mlin (LBMM). Divostin (LBDI). Blagotin (BL) (Serbia)

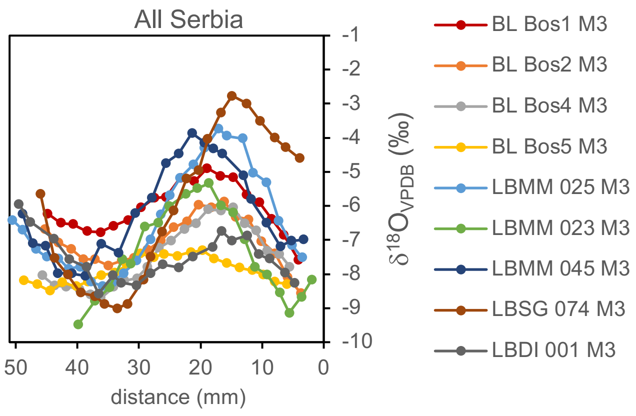

Supplementary Figure S4: Results from the sequential analysis of enamel δ18O values at Těšetice-Kyjovice (Czech Republic)

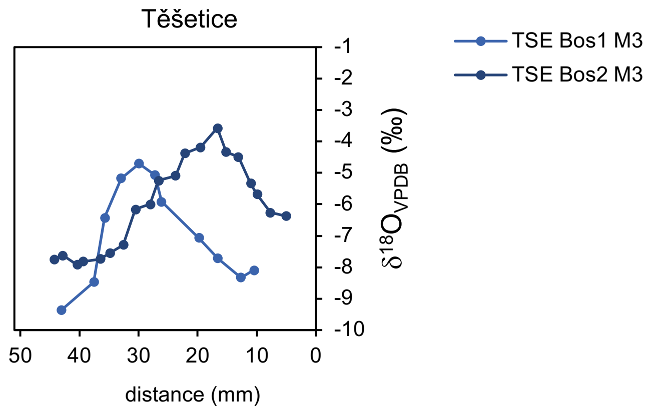

Supplementary Figure S5: Results from the sequential analysis of enamel δ18O values at Borduşani (Romania)

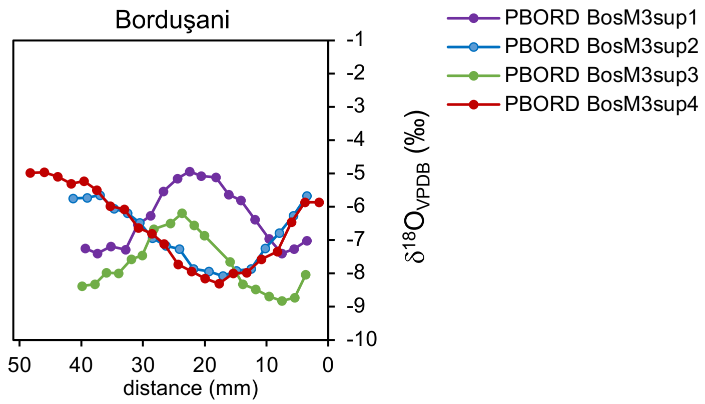

Supplementary Figure S6: Results from the sequential analysis of enamel δ18O values at Auriac (France)

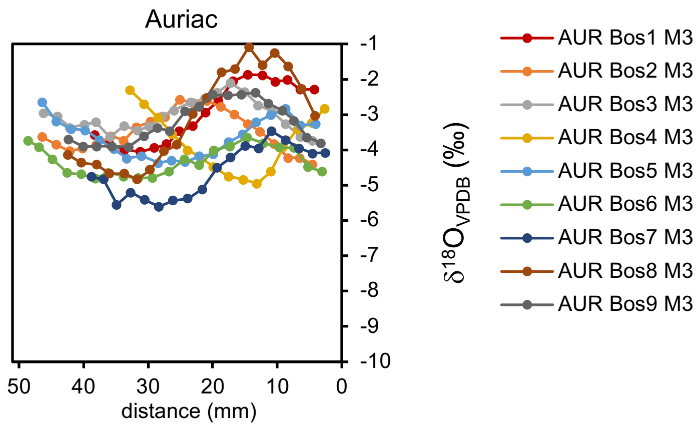

Supplementary Figure S7: Results from the sequential analysis of enamel $\delta^{18}O$ values at Alsónyék (Hungary)

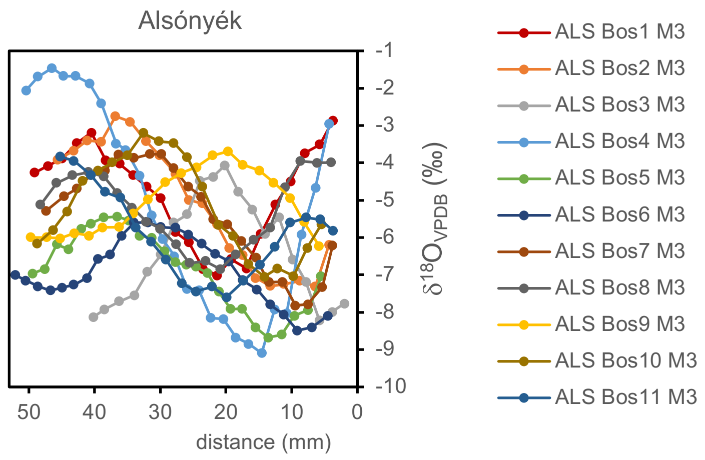

Supplementary Method S1: $\delta^{18}O$ sequences modelling: methodological steps (Picture of the tooth by Marie Balasse).

The modeling uses the equation from Balasse et al. (2012):
$\delta^{18}O_{model} = A \cdot \cos(2\Pi (x - x0)/X) + M$

1- Sequential analysis of enamel $\delta^{18}O$ value, highlighing intra-inter-individual variability

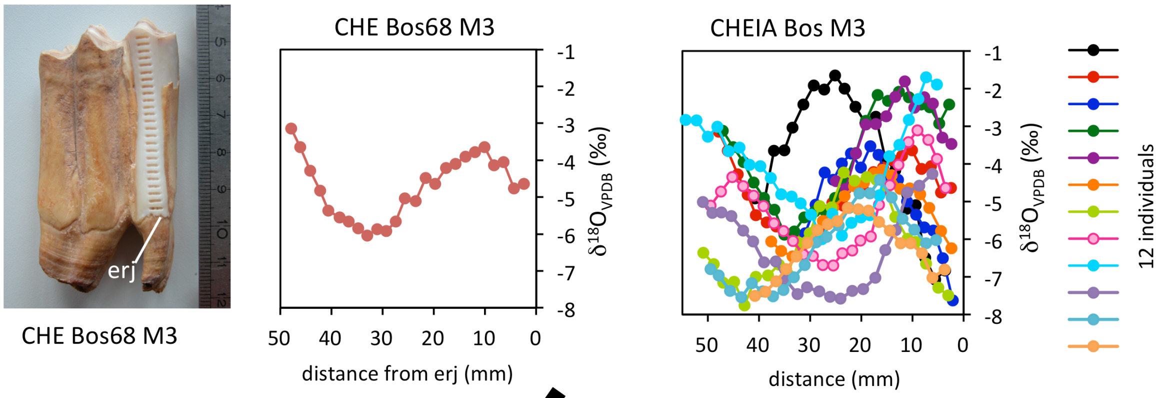

2- Modelling, determination of x_0 and normalisation using X

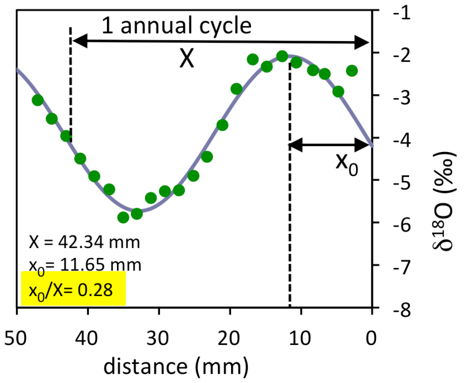

3- The circular representation

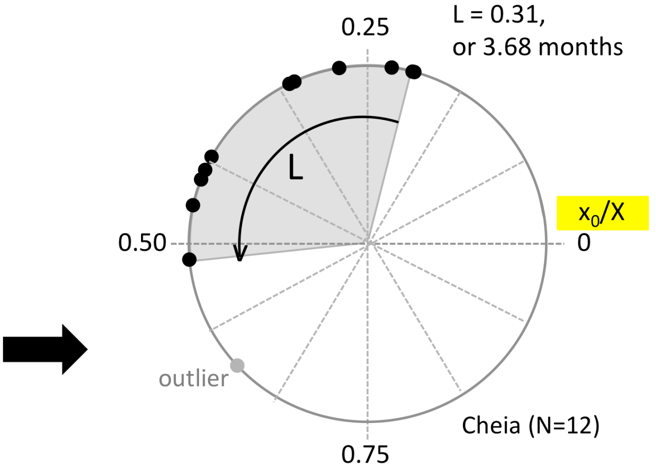

L is the length of the birth period

Balasse, M., Obein, G., Ughetto-Monfrin, J. & Mainland, I. 2012. Investigating seasonality and season of birth in past herds: a reference set of sheep enamel stable oxygen isotope ratios. *Archaeometry* **54**, 349–368.

Supplementary Table S10: Results from the modelling the $\delta^{18}O$ sequences. Calculation of the best fit (method of least squares) between the measured and the modeled dataset for combined variation of X (period), A (amplitude), x_0 (delay) and M (mean).
r = Pearson's correlation coefficient.

Sites (Country)	Specimen ID	Lower/ Upper Left/Right	X (mm)	A (‰)	x_0 (mm)	M (‰)	r Pearson	credit
Starčevo-Grad (Serbia)	LBSG 074 M3	Lower R	44.8	3.00	12.8	-6.00	0.99	This study
Magareći mlin (Serbia)	LBMM 025 M3	Lower L	42.4	2.12	16.9	-6.40	0.97	This study
	LBMM 045 M3	Lower R	40.1	1.86	20.0	-6.05	0.98	This study
	LBMM 023 M3	Lower L	41.1	1.99	21.3	-7.51	0.99	This study
Divostin (Serbia)	LBDI 001 M3	Lower L	31.8	0.75	16.2	-7.63	0.93	This study
Blagotin (Serbia)	BL Bos1 M3	Lower L	36.0	0.86	19.4	-5.92	0.98	This study
	BL Bos2 M3	Lower L	32.7	0.91	18.5	-6.95	0.97	This study
	BL Bos4 M3	Lower L	41.0	1.23	17.3	-7.46	0.99	This study
	BL Bos5 M3	Lower R	41.2	0.51	23.1	-7.86	0.98	This study
Těšetice -Kyjovice	TSE Bos1 M3	Lower L	*Could not be modeled*					This study
(Czech Republic)	TSE Bos2 M3	Lower R	42.6	1.93	18.3	-6.04	0.98	This study
Apc (Hungary)	APC Bos1 M3	Lower R	43.0	2.84	11.5	-5.73	0.99	This study
	APC Bos2 M3	Lower L	40.7	1.39	22.6	-7.37	0.99	This study
	APC Bos3 M3	Lower L	37.1	1.70	33.3	-5.67	0.96	This study
	APC Bos4 M3	Lower L	28.7	1.19	34.3	-5.66	0.96	This study
	APC Bos5 M3	Lower L	45.6	1.83	20.4	-7.21	0.99	This study
	APC Bos6 M3	Lower L	39.4	1.64	15.7	-7.83	0.99	This study
	APC Bos7 M3	Lower R	*Could not be modeled*					This study
	APC Bos8 M3	Lower R	44.5	1.91	6.1	-6.06	0.98	This study
Balatonszárszó (Hungary)	BAL Bos1 M3	Lower L	38.6	2.06	30.8	-5.25	0.97	This study
	BAL Bos2 M3	Lower L	44.8	2.55	13.4	-6.01	0.99	This study
	BAL Bos3 M3	Lower L	44.9	1.41	15.5	-7.10	0.98	This study
	BAL Bos4 M3	Lower R	51.1	2.35	12.8	-4.47	1.00	This study
	BAL Bos5 M3	Lower L	33.6	1.55	28.4	-5.79	0.93	This study
Alsónyék (Hungary)	ALS Bos1 M3	Upper R	39.7	1.69	1.4	-5.05	0.98	This study
	ALS Bos2 M3	Upper R	51.5	2.12	36.9	-5.14	0.99	This study
	ALS Bos3 M3	Upper R	36.0	1.81	20.6	-6.20	0.98	This study
	ALS Bos4 M3	Upper R	48.8	3.55	43.4	-5.24	0.98	This study
	ALS Bos5 M3	Upper R	46.3	1.40	36.8	-6.90	0.98	This study
	ALS Bos6 M3	Upper R	39.6	1.10	28.4	-7.40	0.99	This study
	ALS Bos7 M3	Upper L	49.6	1.83	33.8	-5.50	0.98	This study
	ALS Bos8 M3	Upper R	38.4	1.21	3.0	-5.53	0.99	This study
	ALS Bos9 M3	Upper R	38.7	1.11	21.3	-4.88	0.98	This study
	ALS Bos10 M3	Upper R	40.8	1.80	32.8	-5.05	0.98	This study
	ALS Bos11M3	Upper R	43.7	1.57	0.8	-6.33	1.00	This study

Sites (Country)	Specimen ID	Lower/ Upper Left/Right	X (mm)	A (‰)	x_0 (mm)	M (‰)	r Pearson	credit
Cheia (Romania)	CHE Bos54 M3	Lower L	36.8	1.08	7.7	-4.69	0.94	raw data (1) *
	CHE Bos61 M3	Lower R	38.5	1.22	8.0	-6.49	0.96	raw data (1) *
	CHE Bos80 M3	Lower R	41.3	1.93	25.7	-3.75	0.98	raw data (1) *
	CHE Bos107 M3	Lower R	35.0	1.41	11.1	-3.51	0.96	raw data (1) *
Bordușani (Romania)	PBORD Bos28 M3	Lower L	42.0	1.40	16.1	-5.13	0.99	raw data (2) *
	PBORD Bos29 M3	Lower L	45.0	1.93	13.4	-6.74	0.98	raw data (2) *
	PBORD Bos31 M3	Lower R	46.6	1.21	15.8	-6.04	0.96	raw data (2) *
	PBORD Bos32 M3	Lower L	40.0	1.30	21.3	-6.71	0.99	raw data (2) *
	PBORD Bos35 M3	Lower R	42.3	1.15	19.5	-6.03	0.97	raw data (3) *
	PBORD Bos37 M3	Lower R	36.4	1.08	17.8	-6.06	0.97	raw data (2) *
	PBORD Bos39 M3	Lower R	33.5	1.19	7.9	-6.73	0.98	raw data (2) *
	PBORD Bos41 M3	Lower L	40.9	1.30	11.9	-6.55	0.98	raw data (2) *
	PBORD Bos44 M3	Lower L	38.5	1.45	15.1	-5.92	0.99	raw data (2) *
	PBORD BosM3sup1	Upper L	30.6	1.21	21.1	-6.13	0.99	This study
	PBORD BosM3sup2	Upper L	39.9	1.19	38.3	-6.84	0.98	This study
	PBORD BosM3sup3	Upper L	31.7	1.11	24.4	-7.55	0.97	This study
	PBORD BosM3sup4	Upper R	47.5	1.50	41.9	-6.68	0.99	This study
Hârșova (Romania)	HVA Bos19 M3	Lower L	41.6	1.71	21.6	-6.14	1.00	raw data (2) *
Bercy (France)	BQS Bos3 M3	Lower R	30.4	0.99	7.5	-5.84	0.99	raw data (4) *
Auriac (France)	AUR Bos1 M3	Lower L	39.3	1.10	12.0	-2.99	1.00	This study
	AUR Bos2 M3	Lower L	34.8	0.74	22.7	-3.36	0.97	This study
	AUR Bos3 M3	Lower L	30.6	0.64	18.7	-2.94	0.96	This study
	AUR Bos4 M3	Lower L	*Could not be modeled*					This study
	AUR Bos5 M3	Lower R	38.8	0.66	8.2	-3.75	0.98	This study
	AUR Bos6 M3	Lower R	36.3	0.54	14.4	-4.32	0.96	This study
	AUR Bos7 M3	Lower R	39.2	0.95	9.7	-4.63	0.98	This study
	AUR Bos8 M3	Lower R	42.5	1.77	13.2	-3.14	0.99	This study
	AUR Bos9 M3	Lower L	38.3	0.81	16.9	-3.21	0.98	This study

(1) Balasse *et al.* 2014; (2) Balasse *et al.* 2017; (3) Gillis *et al.* 2013; (4) Balasse *et al.* 2012a
* modelling: this study

Balasse, M., Boury, L., Ughetto-Monfrin, J. & Tresset, A. Stable isotope insights ($\delta^{18}O$, $\delta^{13}C$) into cattle and sheep husbandry at Bercy (Paris, France, 4th millennium BC): birth seasonality and winter leaf foddering. *Environ. Archaeol.* **17 (1)**, 29-44 (2012)

Balasse, M. *et al.* Cattle and sheep herding at Cheia, Romania, at the turn of the fifth millennium cal BC: a view from stable isotope analysis. In *Early Farmers: The View from Archaeology and Science* (eds. Whittle, A. & Bickle, P.) 115–142 (Oxford University Press, 2014)

Balasse, M. *et al.* Investigating the scale of herding in Chalcolithic pastoral communities settled by the Danube river in the 5th millennium BC: a case study at Borduşani-Popină and Hârşova-tell (Romania). *Quat. Int.* **436B**, 29-40 (2017)

Gillis, R. *et al.* Sophisticated cattle dairy husbandry at Borduşani-*Popină* (Romania, fifth millennium BC: the evidence from complementary analysis of mortality profiles and stable isotopes. *World Archaeol.* **45**, 447-472 (2013)

Supplementary Data S1: Results from the isotope analysis of young cattle at Cheia (Romania)

In the feral Chillingham cattle breeding throughout the year, 50% of calves die before maturity, most of them before reaching 12 months, and calf mortality is largely attributable to bad weather (Hall & Hall, 1988). When available, cattle mortality profiles are briefly described in Supplementary Note S1 and were discussed in the original publications. Mortality within the first 12 or 15 months of life (depending on the method used for age attribution) is usually around 15-25 % (details in Supplementary Note S1). Infant mortality cannot be directly compared between sites, due to differences in sample size, methodology and different taphonomic contexts (potentially leading to differential preservation of low density remains). They cannot either be compared to infant and juvenile mortality in feral cattle population, as the domestic status would reduce mortality due to the care given by the herders to females and their offspring around birth time; on the other hand, calf mortality would also result from deliberate slaughter, adding to natural mortality. That being said, it is likely that part of this mortality arose from natural causes. If mortality were higher in calves born out of season, relying on results obtained from the M3 alone could cause out-of-season births to be overlooked. To check for a bias in the estimation of the distribution of births due to the absence of cattle under two years old in the M3 samples, birth seasonality was also investigated in the fourth deciduous premolar (dP4) and the first molar (M1) at Cheia. At Cheia, the 0-6 m and 5-13 m (< stage Ct) age classes (following Jones & Sadler, 2012) include respectively 7 % and 12.8 % of cattle dental remains (N = 360).

The dataset is composed of 15 calves whose age at death, estimated from tooth eruption and wear stages (Jones & Sadler, 2012), is between 0-1 month and 5-13 months. All calves potentially died within their first year, when infantile mortality is highest. The dP4 crown formation is almost completed at birth (Brown *et al*., 1960) and therefore enamel mineralization is partly prenatal in this tooth; although the first molar crown is partly formed at birth, enamel mineralization is delayed (Brown et al., 1960; Balasse, 2002) and mostly occurs within the first year after birth, including the suckling period. Seasonal variations in $\delta^{18}O$ values of the mother cow body water are expectedly passed on to the foetus throughout prenatal life, and seasonal variations in ingested water is shown to be transmitted to the milk (Chen *et al*., 2017). Therefore, the $\delta^{18}O$ records in the dP4 and M1 are expected to reflect the seasonal trends.

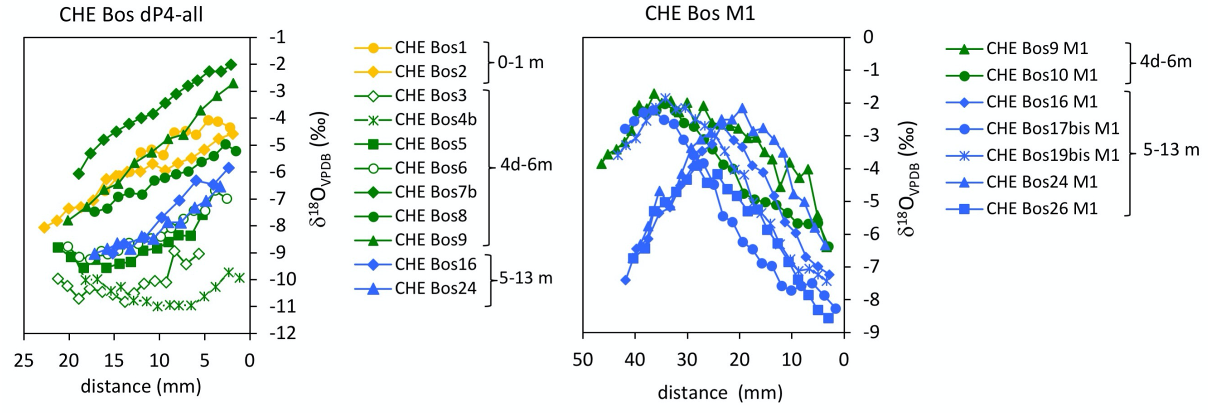

Figure S8: Results from the stable oxygen isotope analysis of the cattle lower dP4 and M1 at Cheia

Eleven calves delivered a lower fourth deciduous premolar (dP4). The intra-tooth variation in $\delta^{18}O$ values is very similar in ten of them, with increasing values along tooth crown (Table S11 and Figure S8), possibly reflecting the transition between winter to summer. Only CHE Bos 4b (aged 4 days-6 months) shows a different pattern with rather stable and low $\delta^{18}O$ values. Seven calves delivered a lower first molar (M1). Among those, the calves aged between 4 days-6 months and 5-18 months show very similar patterns of variation in the $\delta^{18}O$ values, suggesting that they were all born within the same

period of the seasonal cycle. The highest $\delta^{18}O$ values recorded in the earliest (highest) part of the crown may reflect the summer, followed by decreasing values during autumn and towards early winter. In two calves aged 5-13 months (CHE Bos 24 and CHE Bos 26), potentially older (> stage Cc - Complete crown), the maximum $\delta^{18}O$ values are recorded lower in tooth crown. Although inter-individual variability in the tooth size and timing of formation may also create such an offset, this one could suggest they were born 2-3 months earlier compared to the other five calves. Nevertheless, the offset is not obvious in the dP4 of CHE Bos24 (Figure S8).

At Cheia, a restricted birth period was concluded from the analysis conducted in the M3 (Balasse *et al.*, 2014). Overall, among calves dead within their first year, the distribution of births also seems to have been restricted to a short period. In third molar bearing tooth rows (mature cattle), the dP4 shed around two years, is no longer present while much of the stable isotopic record is lost due to wear in the M1. Consequently, it cannot be demonstrated, whether the calves with a dP4 and M1 were born at a similar season as mature cattle providing the lower M3, or at a different season. Calving occurring close in time, but in the tail of the main distribution (earlier, or later than the most favorable period) cannot be precluded. In this case, the under-representation of these births in the M3 record would cause a reduction of the length of the calving period as observed in the M3. Nevertheless, these results do not support in any way the hypothesis of a year-round calving biased by the surviving rate. This approach should be corroborated using data from other assemblages in the future.

Balasse M. 2002. Reconstructing dietary and environmental history from enamel isotopic analysis: time resolution of intra-tooth sequential sampling. *Int. J. Osteoarchaeol*. **12**, 155-165.

Balasse M., Tornero C., Bréhard S., Ughetto-Monfrin J., Voinea V., Bălăşescu A., 2014. Cattle and sheep herding at Cheia, Romania, at the turn of the fifth millennium cal BC: a view from stable isotope analysis. In *Early Farmers: the View from Archaeology and Science* (eds. Whittle, A., Bickle, P.), 115–142, Proc. Br. Acad. Oxford University Press, Oxford.

Brown W.A.B., Christofferson P.V., Massler M., Weiss M.M.B. 1960. Postnatal development in cattle. *Am. J. Vet. Res.* **21 (80)**, 7-34.

Chen G., Schnyder H., Auerswald K. 2017. Model explanation of the seasonal variation of $\delta^{18}O$ in cow (*Bos taurus*) hair under temperate conditions. *Sci. Rep.* **7**, 320. doi:10.1038/s41598-017-00361-y.

Hall, S. J. G. & Hall, J. G. Inbreeding and population dynamics of the Chillingham wild cattle (*Bos taurus*). *J. Zool.* **216**, 479-493 (1988)

Jones G.G., Sadler P. 2012. Age at death in cattle: methods, older cattle and known-age reference material. *Environ. Archaeol.* **17 (1)**, 11-28.

Table S11: Results from the sequential analysis of enamel δ^{18}O values: Cheia (Romania)

**CHE Bos1 dP4**		**CHE Bos2 dP4**		**CHE Bos3 dP4**		**CHE Bos4b dP4**		**CHE Bos5 dP4**		**CHE Bos6 dP4**		**CHE Bos7b dP4**		**CHE Bos8 dP4**		**CHE Bos9 dP4**		**CHE Bos16 dP4**		**CHE Bos24 dP4**	
mm	δ^{18}O (‰)	mm	δ^{18}O (‰)	mm	δ^{18}O (‰)	mm	δ^{18}O (‰)	mm	δ^{18}O (‰)	mm	δ^{18}O (‰)	mm	δ^{18}O (‰)	mm	δ^{18}O (‰)	mm	δ^{18}O (‰)	mm	δ^{18}O (‰)	mm	δ^{18}O (‰)
16.0	-6.71	22.8	-8.06	22.5	-8.38	20.9	-7.40	21.2	-8.81	20.1	-8.78	18.9	-6.06	17.2	-7.48	20.1	-7.80	15.2	-9.03	17.2	-9.03
14.5	-6.13	21.4	-7.81	21.2	-9.97	19.4	-9.15	19.8	-9.17	18.9	-9.16	17.6	-5.31	15.8	-7.35	18.0	-7.30	13.9	-8.67	15.8	-8.86
13.3	-5.99	20.0	-7.36	20.2	-10.24	18.2	-10.02	18.4	-9.57	17.7	-9.24	16.2	-4.81	14.6	-6.92	16.1	-6.66	11.7	-8.46	14.7	-8.66
12.1	-5.28	18.7	-7.30	18.9	-10.70	16.9	-9.98	17.1	-9.27	16.4	-8.94	14.8	-4.50	13.3	-6.78	14.6	-6.43	9.8	-7.70	13.2	-8.86
10.9	-5.17	17.3	-7.05	17.7	-10.33	15.4	-10.42	15.9	-9.56	15.7	-9.06	13.4	-4.22	11.9	-6.84	12.8	-5.67	7.8	-7.05	12.0	-8.41
9.6	-5.38	15.9	-6.26	16.4	-10.45	14.3	-10.27	14.4	-9.41	14.6	-8.92	12.0	-3.99	10.6	-6.32	10.9	-5.27	5.9	-6.32	10.7	-8.49
8.4	-4.53	14.8	-6.16	15.2	-10.55	12.9	-10.77	13.2	-9.34	13.4	-8.64	10.7	-3.84	9.4	-6.22	9.1	-4.77	4.0	-6.47	9.1	-7.87
7.1	-4.48	13.6	-5.99	13.8	-10.82	11.4	-10.81	11.8	-8.93	12.4	-8.67	9.4	-3.44	8.2	-6.08	7.4	-4.62	2.4	-5.85	7.7	-7.87
5.9	-4.62	12.1	-5.98	12.8	-10.51	10.2	-10.99	10.3	-8.83	10.9	-8.46	8.2	-3.10	6.8	-5.98	5.5	-3.72			6.1	-7.32
4.6	-4.08	10.8	-5.70	11.7	-10.14	8.9	-10.93	9.0	-8.61	9.9	-8.40	6.9	-2.80	5.4	-5.62	3.7	-3.17			4.9	-7.07
3.5	-4.12	9.4	-5.95	10.5	-10.05	7.9	-10.95	7.9	-8.36	8.7	-8.08	5.8	-2.60	4.0	-5.41	1.8	-2.71			3.3	-6.54
2.2	-4.34	8.0	-5.67	9.3	-10.10	6.5	-10.95	6.6	-8.37	7.3	-7.75	4.5	-2.26	2.7	-4.98						
		6.5	-5.48	8.3	-8.94	5.1	-10.63	5.3	-7.59	6.2	-7.44	3.2	-2.27	1.5	-5.22						
		5.0	-5.17	7.1	-9.42	3.8	-10.27			5.0	-7.44	2.1	-2.01								
		3.4	-4.76	5.6	-9.04	2.4	-9.73			3.9	-6.69										
		1.9	-4.58			1.2	-9.94			2.6	-6.98										

Table S11 (continued): Results from the sequential analysis of enamel $\delta^{18}O$ values: Cheia (Romania)

CHE Bos9 M1		CHE Bos10 M1		CHE Bos16 M1		CHE Bos17b M1		CHE Bos19b M1		CHE Bos24 M1		CHE Bos26 M1	
mm	$\delta^{18}O$ (‰)	mm	$\delta^{18}O$ (‰)	mm	$\delta^{18}O$ (‰)	mm	$\delta^{18}O$ (‰)	mm	$\delta^{18}O$ (‰)	mm	$\delta^{18}O$ (‰)	mm	$\delta^{18}O$ (‰)
46.5	-3.85	39.6	-2.25	41.8	-7.40	42.0	-2.79	43.3	-3.59	40.5	-6.72	40.4	-6.73
45.2	-3.56	37.9	-2.35	39.8	-6.45	40.2	-2.56	41.7	-3.29	39.0	-6.31	38.1	-6.44
43.6	-3.41	35.9	-2.22	37.6	-6.14	38.4	-2.30	39.9	-3.08	37.5	-5.76	36.4	-5.30
42.0	-3.19	34.3	-2.04	35.4	-5.37	36.6	-2.23	37.9	-2.54	35.4	-4.68	34.3	-5.03
40.7	-2.85	32.4	-2.24	33.3	-5.18	34.5	-2.52	36.1	-2.14	33.3	-5.12	32.1	-4.71
39.3	-2.08	30.7	-2.62	31.3	-4.52	32.6	-2.64	34.2	-1.85	31.1	-4.15	30.2	-4.33
37.7	-2.18	28.7	-2.72	29.0	-3.95	30.8	-3.12	32.2	-2.20	29.3	-3.37	28.2	-3.91
36.4	-1.72	26.9	-3.11	27.2	-3.47	28.9	-3.59	30.4	-2.15	27.5	-3.11	26.3	-4.43
34.9	-2.01	25.2	-3.42	25.3	-3.27	27.0	-3.84	28.5	-2.49	25.6	-2.90	24.3	-4.18
33.2	-1.94	23.1	-3.88	23.4	-2.67	24.9	-4.50	26.6	-2.70	23.5	-2.51	22.4	-4.63
31.6	-2.28	21.3	-4.01	21.1	-3.14	23.2	-5.46	24.8	-2.98	21.5	-2.51	20.6	-4.83
30.1	-1.99	19.2	-4.76	19.5	-3.34	21.4	-5.63	22.9	-3.47	19.5	-2.16	18.4	-5.30
28.6	-2.61	17.2	-4.94	17.6	-3.92	19.4	-6.24	21.0	-4.03	17.4	-2.86	16.5	-5.24
26.9	-2.09	15.1	-5.03	15.5	-4.11	17.5	-6.47	19.2	-4.19	15.5	-2.77	14.7	-5.87
25.3	-2.61	13.0	-5.11	13.4	-4.84	15.7	-6.90	17.4	-5.04	13.5	-3.12	12.8	-6.28
23.6	-2.66	10.9	-5.36	11.4	-5.63	13.8	-6.97	15.8	-5.36	11.7	-3.51	10.6	-6.85
21.9	-2.70	8.9	-5.68	9.3	-5.96	12.0	-7.58	14.1	-5.68	9.7	-4.77	8.8	-7.39
20.3	-2.78	6.8	-5.68	7.3	-6.69	10.1	-7.72	12.3	-6.31	7.6	-5.02	6.8	-7.86
18.5	-3.06	5.0	-5.66	5.1	-6.99	8.2	-7.58	10.3	-6.77	5.7	-5.80	5.1	-8.31
16.8	-3.05	3.0	-6.37	2.8	-7.23	6.1	-7.50	8.8	-7.12	3.6	-6.34	3.0	-8.55
15.3	-3.50					3.8	-7.87	6.7	-7.06				
13.8	-3.71					1.6	-8.27	5.0	-7.23				
12.2	-4.55							3.3	-7.43				
10.5	-3.82												
8.5	-4.28												
6.9	-4.02												
5.1	-5.42												
3.4	-6.39												

Supplementary Data S2: Osteometric assessment of the status of the teeth included in the study

The selected third molars of Bos (cattle/aurochs) were assessed using morphometrical criteria.

Lower third molars

For the lower M3 (Table S12 and Figs. S9 &10), the antero-posterior diameter (DAP or length) and the transversal diameter (DT or breadth) at neck (Ducos 1968) were compared to measurements reported for Holocene aurochs from Denmark (Degerbøl and Fredskild, 1970) and Mesolithic aurochs from La Montagne (Sénas, southeastern France, Helmer & Monchot, 2006). Additionally, the data from the Middle Neolithic in France and the Romanian Chalcolithic (Fig. S10) is compared to measurements taken on the largest bovine molars from the Gumelnita levels at Vitănești (N=13) and Borduşani (N=1), even though the status of these teeth, identified from morphometric criteria alone as aurochs, is not certain (Table S13).

For the Early Neolithic (Fig. S9), one tooth from Magarući mlin (LBMM025), one tooth from Ludwinowo (LUDBos10; not included in the final dataset because its $\delta^{18}O$ sequence could not be modeled), and two teeth from Bishoffsheim (BISBos1 and Bos2) fall outside of the range defined in the Denmark aurochs, but are very close in size to the smallest aurochs at La Montagne. One tooth from Chotěbudice (CHOBos1) falls within the range defined for aurochs. The status of these specimens is therefore uncertain. It must be noted however, that the aurochs from Denmark and southwestern Europe used here as reference were, on average, smaller than those from Central Europe (discussion and references in Berthon *et al.*, 2018).

The measurements taken on the lower M3 from the Middle Neolithic and Chalcolithic sites (Cheia, Borduşani, Hârşova, Bercy and Auriac) do not overlap with those obtained from aurochs (Figure S10).

Upper third molars

Measurements of aurochs upper molars are very scarce in the published literature. The upper M3s from Borduşani and Alsónyék (Table S14) were compared to the Durankulak dataset. The latter includes measurements of the length of the M3 on domestic cattle (N=10) and presumed aurochs (N=3) from the Varna II and Late Bronze Age levels (Manhart, 1998). Although it is difficult to determine from this reduced dataset the lower threshold value for the aurochs, we observe that the upper molars from Borduşani, Măgura and Alsónyék are all smaller than those from the Durankulak aurochs (Fig. S11). Nevertheless, a doubt may remain for ALSBos9 and ALSBOS11 from Alsónyék, which delivered values (≥ 34mm) outside of the range defined on cattle, although also lower than those defined for the presumed aurochs (Fig. S11).

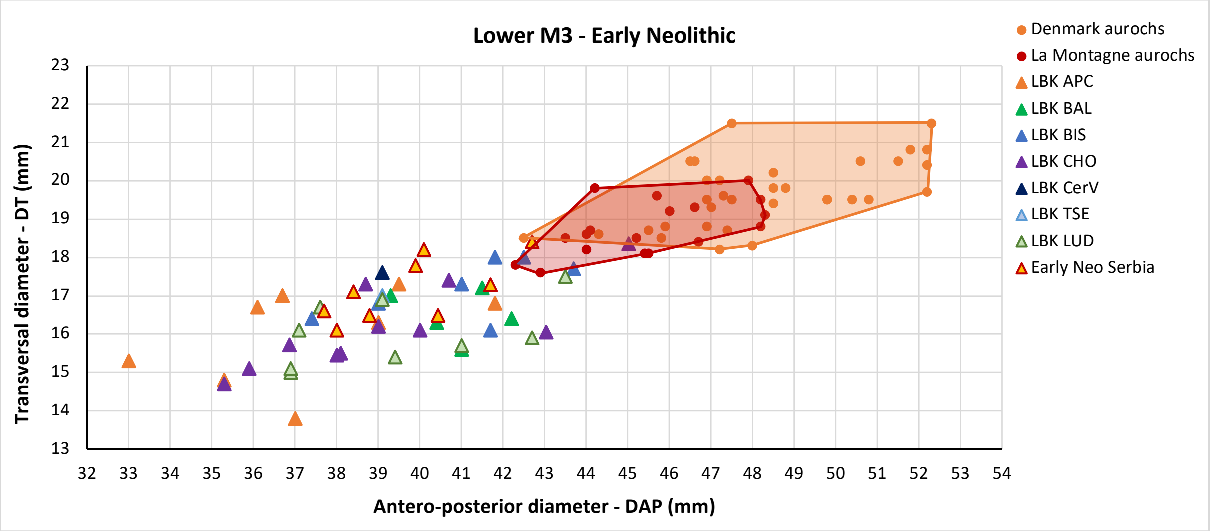

Figure S9: Measurements taken on the *Bos* lower M3 at the early Neolithic sites, and comparison with the Holocene aurochs from Denmark (Degerbøl & Fredskild, 1970) and Mesolithic aurochs from France (Helmer & Monchot, 2006).

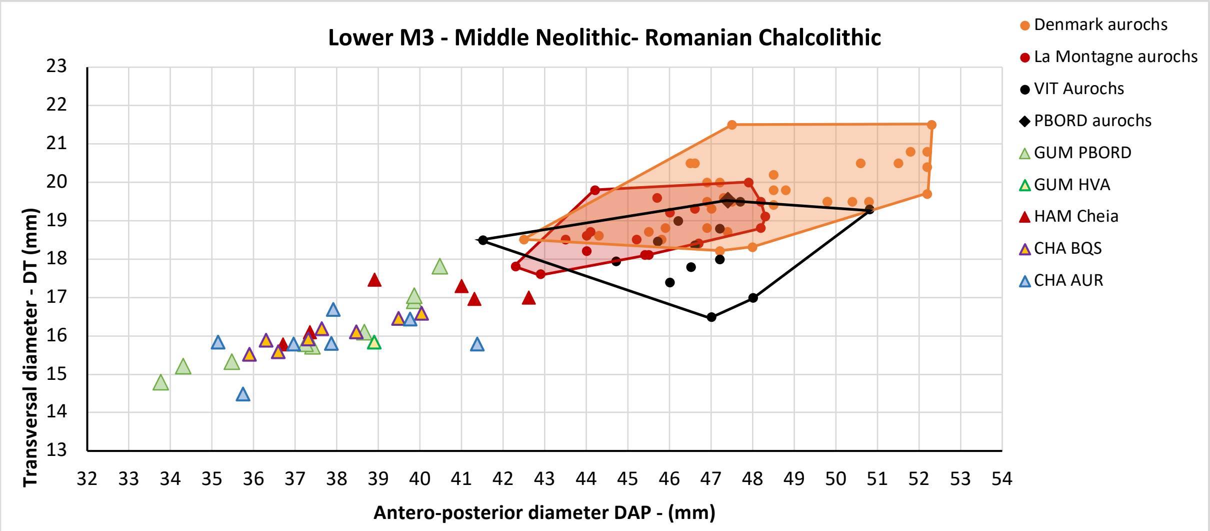

Figure S10: Measurements taken on the *Bos* lower M3 at the Middle Neolithic sites in France and Chalcolithic sites in Romania and comparison with the Holocene aurochs from Denmark (Degerbøl & Fredskild, 1970), Mesolithic aurochs from France (Helmer & Monchot, 2006) and possible aurochs from Romania from the Chalcolithic period (this study, Table SM5B).

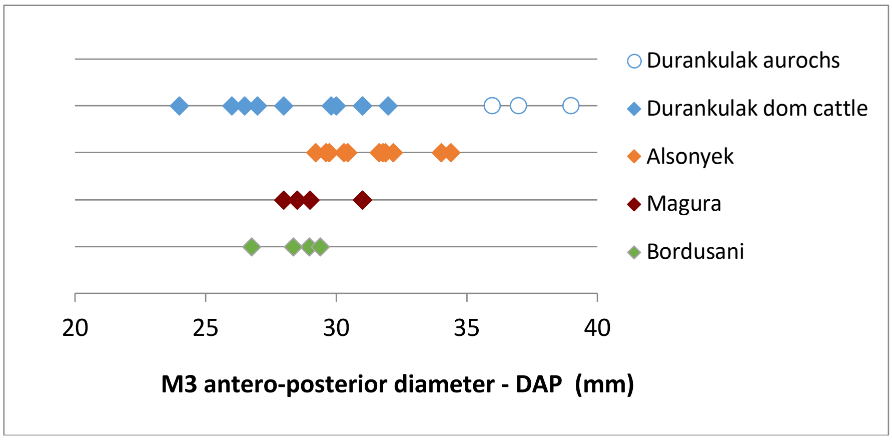

Figure S11: Measurements taken on the *Bos* upper third molars (M3) from Borduşani, Măgura and Alsónyék, compared to the Durankulak dataset (Manhart 1988).

References:

Berthon R., Kovačiková L. Tresset A., Balasse M. 2018. Integration of Linearbandkeramik cattle husbandry in the forested landscape of the mid-Holocene climate optimum: Seasonal-scale investigations in Bohemia. *J. Anthropol. Archaeol.* **51**,16-27.

Degerbøl, M., Fredskild, B. 1970. The urus (*Bos primigenius* Bojanus) and Neolithic domesticated cattle (*Bos taurus domesticus* Linné) in Denmark. Det Kongelige Danske Videnskabernes Selskab Biologiske Skrifter **17**, 1-177.

Ducos, P. 1968. *L'origine des animaux domestiques en Palestine*. Mémoire 6. Publications de l'Institut de Préhistoire de l'Université de Bordeaux, Bordeaux.

Helmer, D., Monchot, H. 2006. Un site mésolithique de chasse à l'aurochs (La Montagne, Sénas, Bouches-du-Rhône). *Anthropozoologica* **41**, 215-228.

Manhart, H. 1998. Die vorgeschichtliche Tierwelt von Koprivets und Durankulak und anderen prähistorichen Fundplätzen in Bulgarien aufgrund von Knochenfunden aus archäologischen Ausgrabungen. *Documenta Naturae* **116**, 1–35

Tooth Code	DAP (mm)	DT (mm)	Tooth Code	DAP (mm)	DT (mm)	Tooth Code	DAP (mm)	DT (mm)
BIS1	42.5	18.0	CHO Bos 1	45.0	18.4	PBORD Bos 41	40.5	17.8
BIS2	43.7	17.7	CHO Bos 2	43.0	16.1	PBORD Bos 44	39.9	17.0
BIS3	39.0	16.8	CHO Bos 3	35.3	14.7	PBORD Bos 39	34.3	15.2
BIS4	37.4	16.4	CHO Bos 4	38.0	15.4	PBORD Bos 37	33.8	14.8
BIS5	41.0	17.3	CHO Bos 5	36.9	15.7	PBORD Bos 32	38.7	16.1
BIS6	41.8	18.0	CHO Bos 6	38.7	17.3	HVA Bos 19	38.9	15.8
BIS7	41.7	16.1	CHO Bos 7	40.0	16.1	CHE Bos 76	42.6	17.0
LUD1*	41.0	15.7	CHO Bos 8	38.1	15.5	CHE Bos 66	36.7	15.8
LUD2	39.1	16.9	CHO Bos 9	39.0	16.2	CHE Bos 61	41.3	17.0
LUD3	39.4	15.4	CHO Bos 10	40.7	17.4	CHE Bos 54	41.0	17.3
LUD4	/	16.7	CerV Bos 1*	35.9	15.1	CHE Bos 55	37.4	16.1
LUD5*	37.6	16.7	CerV Bos 2*	/	16.4	CHE Bos 68	38.9	17.5
LUD6	36.9	15.0	CerV Bos 3	39.1	17.6	BQS Bos 1	37.6	16.2
LUD7	36.9	15.1	CerV Bos 4*	35.4	16.0	BQS Bos 2	30.0	16.1
LUD8	37.1	16.1	LBDI001	38.4	17.1	BQS Bos 3	37.3	15.9
LUD9	42.7	15.9	LBMM023	38.0	16.1	BQS Bos 4	36.3	15.9
LUD10*	43.5	17.5	LBMM025	42.7	18.4	BQS Bos 5	35.9	15.5
APC1	36.1	16.7	LBMM045	40.1	18.2	BQS Bos 6	39.5	16.5
APC2	36.7	17.0	LBSG074	37.7	16.6	BQS Bos 7	40.0	16.6
APC3	39.0	16.3	BL Bos1 M3	39.9	17.8	BQS Bos 8	38.5	16.1
APC4	35.3	14.8	BL Bos2 M3	38.8	16.5	BQS Bos 9	36.6	15.6
APC5	41.8	16.8	BL Bos4 M3	/	17.4	AUR Bos 1	37.0	15.8
APC6	33.0	15.3	BL Bos5 M3	41.7	17.3	AUR Bos 2	37.9	15.8
APC7*	37.0	13.8	BL Bos6 M3	40.4	16.5	AUR Bos 3	35.2	15.8
APC8	39.5	17.3	TES1*	37.4	16.5	AUR Bos 4*	37.9	16.7
BAL1	41.5	17.2	TES2	39.1	17.0	AUR Bos 5	/	16.2
BAL2	40.4	16.3	PBORD Bos 31	37.4	15.7	AUR Bos 6	/	15.1
BAL3	41.0	15.6	PBORD Bos 29	35.5	15.3	AUR Bos 7	39.8	16.4
BAL4	39.3	17.0	PBORD Bos 35	39.9	16.9	AUR Bos 8	35.7	14.5
BAL5	42.2	16.4	PBORD Bos 28	37.2	15.8	AUR Bos 9	41.4	15.8

Table S12: Measurements taken on the *Bos* lower M3. DAP: antero-posterior length; DT transversal breadth. BIS: Bischoffsheim ; LUD : Ludwinowo; APC: Apc; BAL: Balatonszárszó; CHO: Chotěbudice; CerV: Černý Vůl; LBSG: Starčevo-Grad; LBMM: Magarεći mlin; LBDI: Divostin; BL: Blagotin; TSE: Těšetice-Kyjovice; PBORD: Borduşani; HVA: Hârşova; CHE: Cheia; BQS: Bercy; AUR: Auriac. (*) the $\delta^{18}O$ sequence could not be modeled, specimen not included in the final stable isotope dataset.

Tooth Code	DAP (mm)	DT (mm)
Vitănești Gum B1	48	17
Vităneștii Gum B1	47	16.5
Vitănești Gum B1	41.5	18.5
Vitănești Gum B1	46.2	19
Vitănești Gum A2	47.2	18
Vitănești Gum A2	47.2	18.8
Vitănești Gum A2	47.7	19.5
Vitănești Gum A2	46	17.4
Vitănești Gum A2	46.5	17.8
Vitănești Gum A2	50.8	19.3
VIT Bos M3	46.6	18.36
VIT Bos 33 M3	45.7	18.46
VIT Bos 36 M3	44.7	17.94
PBORD Bos MB3	47.4	19.5

Table S13: Measurements taken on the lower M3 of large Bos specimens (aurochs?). DAP: antero-posterior length; DT transversal breadth. VIT: Vitănești, Gumelnița A2 and B1 culture. PBORD: Borduşani.

tooth code	length (mm)	comment
PBORD Bos M3 sup1	29.0	
PBORD Bos M3 sup2	26.8	
PBORD Bos M3 sup3	28.4	
PBORD Bos M3 sup4	29.4	
ALS Bos 1 M3sup	29.6	
ALS Bos 2 M3sup	30.3	
ALS Bos 3 M3sup	31.6	
ALS Bos 4 M3sup	30.5	
ALS Bos 5 M3sup	29.7	
ALS Bos 6 M3sup	32.2	
ALS Bos 7 M3sup	31.8	
ALS Bos 8 M3sup	29.2	
ALS Bos 9 M3sup	34.4	
ALS Bos 10 M3sup	31.9	
ALS Bos 11 M3sup	34.0	
MAG Bos1 M3 sup	31.0	measured on picture
MAG Bos2 M3 sup	31.0	measured on picture
MAG Bos3 M3sup	29.0	measured on picture
MAG Bos4 M3sup	28.0	measured on picture
MAG Bos5 M3sup	28.5	measured on picture
Varna II	31.0	
Varna II	31.0	
Varna II	30.0	
Varna II	29.8	
Varna II	37.0	aurochs
Late Bronze Age	26.0	
Late Bronze Age	24.0	
Late Bronze Age	28.0	
Late Bronze Age	26.5	
Late Bronze Age	27.0	
Late Bronze Age	32.0	
Late Bronze Age	36.0	aurochs
Late Bronze Age	39.0	aurochs

Table S14: Measurements taken on the *Bos* upper M3s. The data from the Late Bronze Age and Varna II levels at Durankulak are from Manhart (1988). PBORD: Borduşani. ALS: Alsónyék. MAG: Măgura.

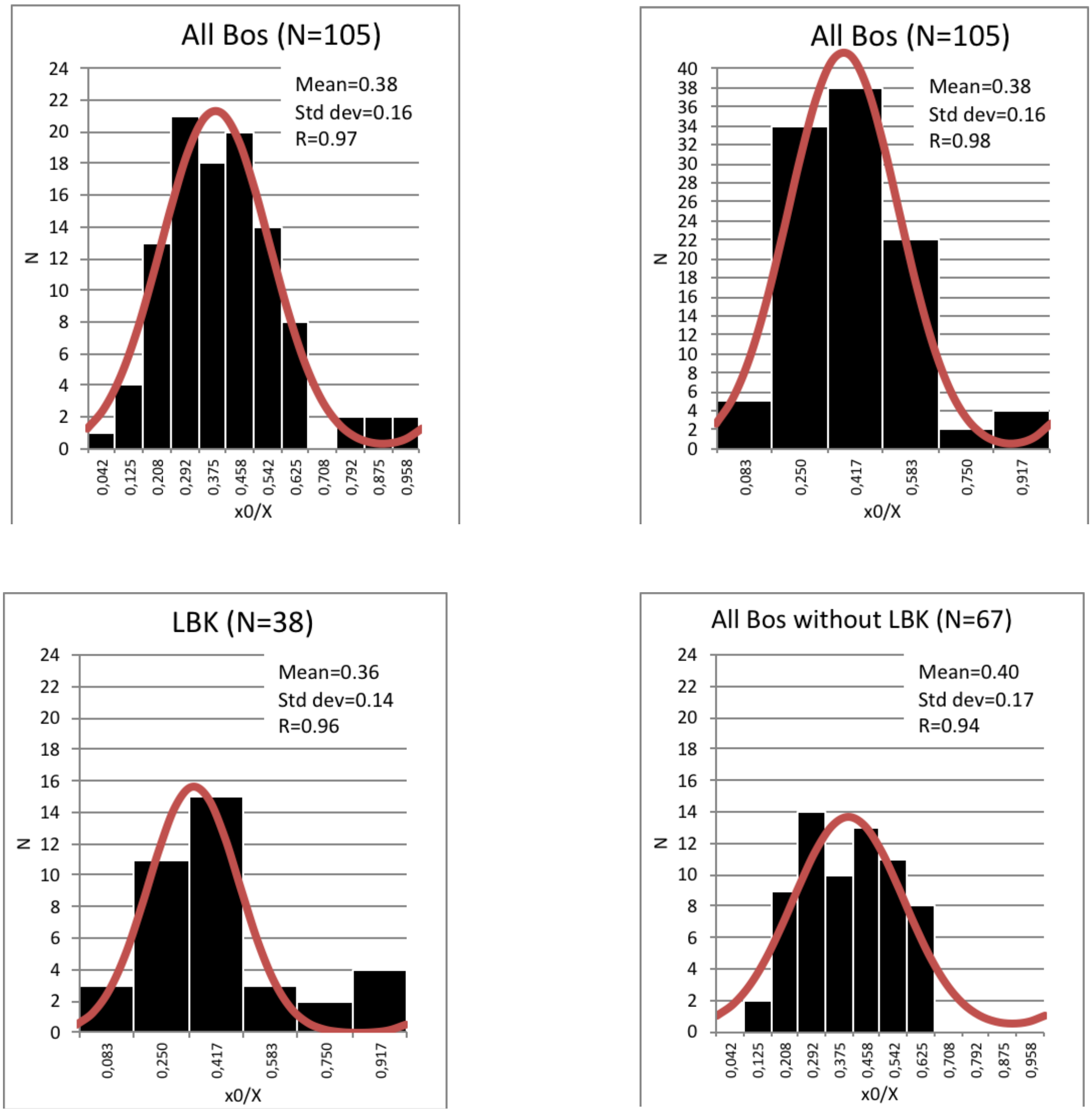

Supplementary Figure S12: Histograms showing the distribution of cattle births when combining all data (All Bos), without LBK data (All Bos without LBK) or considering LBK cattle only (LBK). Results from gaussian curve fitting, correlation coefficient (R).

68% confidence interval without outliers							
	N	start (rad)	end (rad)	start (°)	end (°)	duration (°)	duration (months)
All Serbia	9	2.4	3.6	140	208	68	2.3
CHO & CerV & TSE	10	2.0	3.0	114	170	56	1.9
LUD	6	1.3	3.1	76	176	100	3.3
BIS	6	1.4	3.2	78	182	104	3.5
APC	6	1.0	3.2	57	181	124	4.1
BAL	3	1.5	2.3	86	129	43	1.4
CHE	11	1.5	2.9	85	165	80	2.7
PBORD & HVA	10	1.8	3.1	104	180	76	2.5
BQS	9	1.7	3.7	98	210	112	3.8
AUR	8	1.3	3.4	75	197	122	4.1
MAG (uncor upper)	4	-2.7	-0.4	-154	-23	131	4.4
MAG (cor upper)	4	0.9	3.2	51	182	131	4.4
ALS (uncor upper)	10	-2.5	-0.4	-145	-22	123	4.1
ALS (cor upper)	10	1.1	3.2	60	182	122	4.1

Table S15: Results from the calculation of the main calving peak (68% of births) at individual sites or on a regional scale. All Serbia = Starčevo-Grad. Magareći mlin, Divostin, Blagotin; CHO: Chotěbudice; CerV: Černý Vůl; TSE: Těšetice-Kyjovice; LUD: Ludwinowo; BIS: Bischoffsheim; APC: Apc; BAL: Balatonszárszó-Kis-erdei-dűlő; CHE: Cheia; PBORD: Borduşani; HVA: Hârşova; BQS: Bercy; AUR: Auriac; MAG: Măgura; ALS: Alsónyék.

	**95% confidence interval**						
	N	start (rad)	end (rad)	start (°)	end (°)	duration (°)	duration (months)
All Bos	105	0.3	4.6	17	261	244	8.1
All Bos without LBK	67	0.9	4.2	54	241	187	6.2
LBK Bos only	38	-0.4	4.7	-25	272	297	9.9

	**68% confidence interval without outliers**						
	N	start (rad)	end (rad)	start (°)	end (°)	duration (°)	duration (months)
All Bos	99	1.6	3.2	92	186	94	3.1
All Bos without LBK	67	1.8	3.4	101	194	93	3.1
LBK Bos only	34	1.2	3.1	70	176	106	3.5

Table S16: Results from the calculation of the 95% and 68% confidence intervals for cattle births on the European scale.

Supplementary Method S2: Assessment of the comparability of results obtained on the upper and lower M3.

The sampling was conducted in the lower third molar, except at Măgura and Alsónyék where upper M3s were chosen because they occurred in higher numbers. At both sites, the cattle birth distribution is radically different from those at all other sites, suggesting a shift in the record between the upper and lower M3. In order to investigate the comparability of results obtained on the upper and lower M3, four additional upper M3s were analysed at Borduşani and the results were compared to those obtained from ten lower M3s (nine from Borduşani and one from the neighbouring site of Hârşova). The results are shown in Table S15 and on Figure S13. When comparing the average x_0/X ratio obtained on the lower and upper M3s, a 0.43 (or 5.2 months) difference is found. This correction was applied to the results obtained on the upper M3s at Măgura and Alsónyék (Figure S14). This correction, acquired on a limited number of individuals, must be refined in the future.

**Specimen**	**L/U**	**x_0**	**X**	**x_0/X**
PBORD Bos39 M3	lower	7.9	33.5	0.24
PBORD Bos41 M3	lower	11.9	40.9	0.29
PBORD Bos29 M3	lower	13.4	45.0	0.30
PBORD Bos31 M3	lower	15.8	46.6	0.34
PBORD Bos28 M3	lower	16.1	42.0	0.38
PBORD Bos44 M3	lower	15.1	38.5	0.39
PBORD Bos35 M3	lower	19.5	42.3	0.46
PBORD Bos37 M3	lower	17.8	36.4	0.49
HVA Bos19 M3	lower	21.6	41.6	0.52
PBORD Bos32 M3	lower	21.3	40.0	0.53
Mean lower (± 1σ)				0.39 (± 0.10)
PBORD BosM3sup1	upper	21.1	30.6	0.69
PBORD BosM3sup3	upper	24.4	31.7	0.77
PBORD BosM3sup4	upper	41.9	47.5	0.88
PBORD BosM3sup2	upper	38.3	39.9	0.96
Mean upper (± 1σ)				0.83 (± 0.12)
Difference upper-lower				0.43

Table S17: Comparison of the results obtained from lower and upper M3 at Borduşani (PBORD) and Hârşova (HVA).

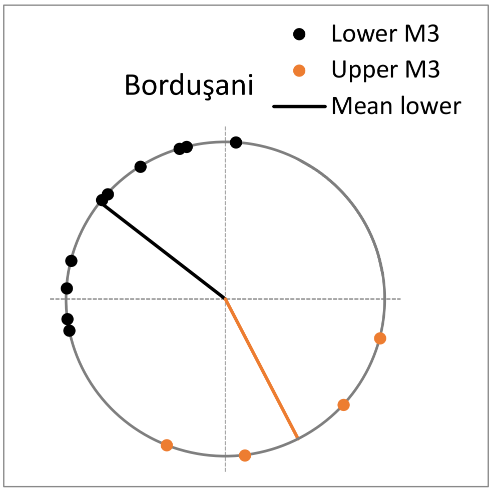

Figure S13: Comparison of the results obtained from upper and lower M3 at Bordușani.

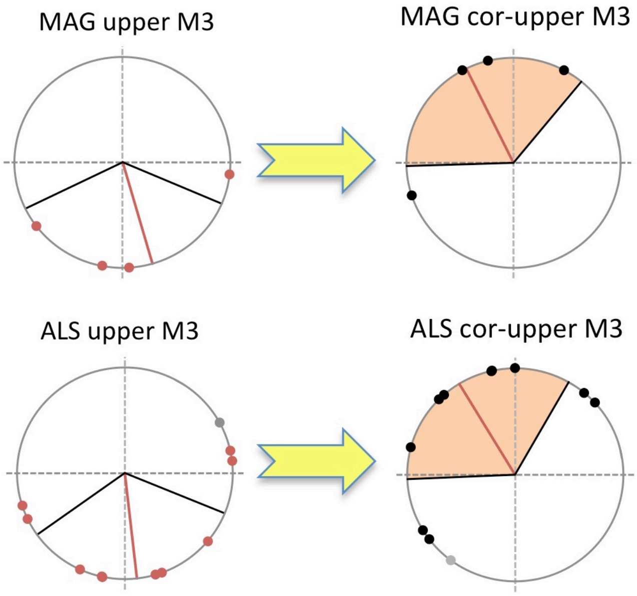

Figure S14: Correction of the results obtained on upper M3s at Măgura (MAG) and Alsónyék (ALS), used for comparison with the sampled lower M3s from other sites.
